# The Context-Dependence of Mutations: A Linkage of Formalisms

**DOI:** 10.1371/journal.pcbi.1004771

**Published:** 2016-06-23

**Authors:** Frank J. Poelwijk, Vinod Krishna, Rama Ranganathan

**Affiliations:** 1 Green Center for Systems Biology, University of Texas Southwestern Medical Center, Dallas, Texas, United States of America; 2 Green Center for Systems Biology and Departments of Biophysics and Pharmacology, University of Texas Southwestern Medical Center, Dallas, Texas, United States of America; National Cancer Institute, United States of America and Tel Aviv University, Israel, UNITED STATES

## Overview

Defining the extent of epistasis—the nonindependence of the effects of mutations—is essential for understanding the relationship of genotype, phenotype, and fitness in biological systems. The applications cover many areas of biological research, including biochemistry, genomics, protein and systems engineering, medicine, and evolutionary biology. However, the quantitative definitions of epistasis vary among fields, and its analysis beyond just pairwise effects remains problematic in general. Here, we bring together a number of previous results that show that different definitions of epistasis are versions of a single mathematical formalism—the weighted Walsh-Hadamard transform. We demonstrate that one of the definitions, the background-averaged epistasis, may be the most informative for describing the epistatic structure of a biological system. Key issues are the choice of effective ensembles for averaging and to practically contend with the vast combinatorial complexity of mutations. In this regard, we discuss strategies for optimally learning the epistatic structure of biological systems.

## Introduction

There has been much recent interest in the prevalence of epistasis in the relationships between genotype, phenotype, and fitness in biological systems [1–7]. Epistasis here is defined as the nonindependence (or context-dependence) of the effect of a mutation, which is a generalization of Bateson’s original definition of epistasis as a genetic interaction in which a mutation “masks” the effect of variation at another locus [8]. It is also in line with Fisher’s broader definition of “epistacy” [9]. Epistasis limits our ability to predict the function of a system that harbors several mutations, given knowledge of the effects of those mutations taken independently [10–13], and makes these relationships increasingly more complex [14–19]. From an evolutionary perspective, the presence of epistatic interactions may limit or entirely preclude trajectories of single-mutation steps towards peaks in the fitness landscape [20–29]. With regard to human health, epistasis complicates our understanding of the origin and progression of disease [30–37]. Thus, interest in the extent of epistatic interactions in biological systems has originated from the fields of protein biochemistry, protein engineering, medicine, systems biology, and evolutionary biology alike.

Originally, epistasis was considered in the context of two genes, but we can define it more broadly as the nonindependence of mutational effects in the genome, whether the effects are within, between, or even outside protein coding regions (e.g., in regulatory regions). The perturbations may go beyond point mutagenesis, but we limit the discussion here for clarity of presentation. Importantly, the definition of epistasis can be extended beyond pairwise effects to comprise a hierarchy of three-way, four-way, and higher-order terms that represent the complete theoretical description of epistasis between the parts that make up a biological system.

How can we quantitatively assign an epistatic interaction given experimentally determined effects of mutations? Because epistasis is deviation from independence, it is crucial to first explicitly state the null hypothesis—asserting what exactly it means to have independent contributions of mutations. This by itself is typically nontrivial. In some cases the phenotype is directly related to a thermodynamic state variable, and the issue is straightforward: independence implies additivity in the state variable. For example, for equilibrium binding reactions between two proteins, independence means additivity in the free energy of binding Δ*G*_bind_, such that the energetic effect of a double mutation is the sum of the energetic effects of each single mutation taken independently. However, in general, many phenotypes cannot be so directly linked to a thermodynamic state variable, and quantification of epistasis needs to be accompanied by a proper rationale for the choice of null hypothesis. In what follows, we will assume this step has already been carried out and we will equate independence with additivity of mutational effects. Epistasis between two mutations is then defined as the degree to which the effect of both mutations together differs from the sum of the effects of the single mutations.

In this paper, we describe three theoretical frameworks that have been proposed for characterizing the epistasis between components of biological systems; these frameworks originate in different fields and use seemingly different calculations to describe the nonindependence of mutations [2,14,24,33,38–46]. We extend previous observations [47–50] to show that these formalisms are different manifestations of a common mathematical principle, which explains their conceptual similarities and distinctions. Each of these formalisms has its value depending on depth of coverage and nature of sampling in the experimental data and the objective of the analysis. In the end, the fundamental issue is to develop practical approaches for optimally learning the epistatic structure of biological systems in the face of the explosive combinatorial complexity of possible epistatic interactions between mutations. Understanding the mathematical relationships between the different frameworks for analyzing epistasis is a key step in this process.

## Results

### Basic definitions

**Fig 1:**
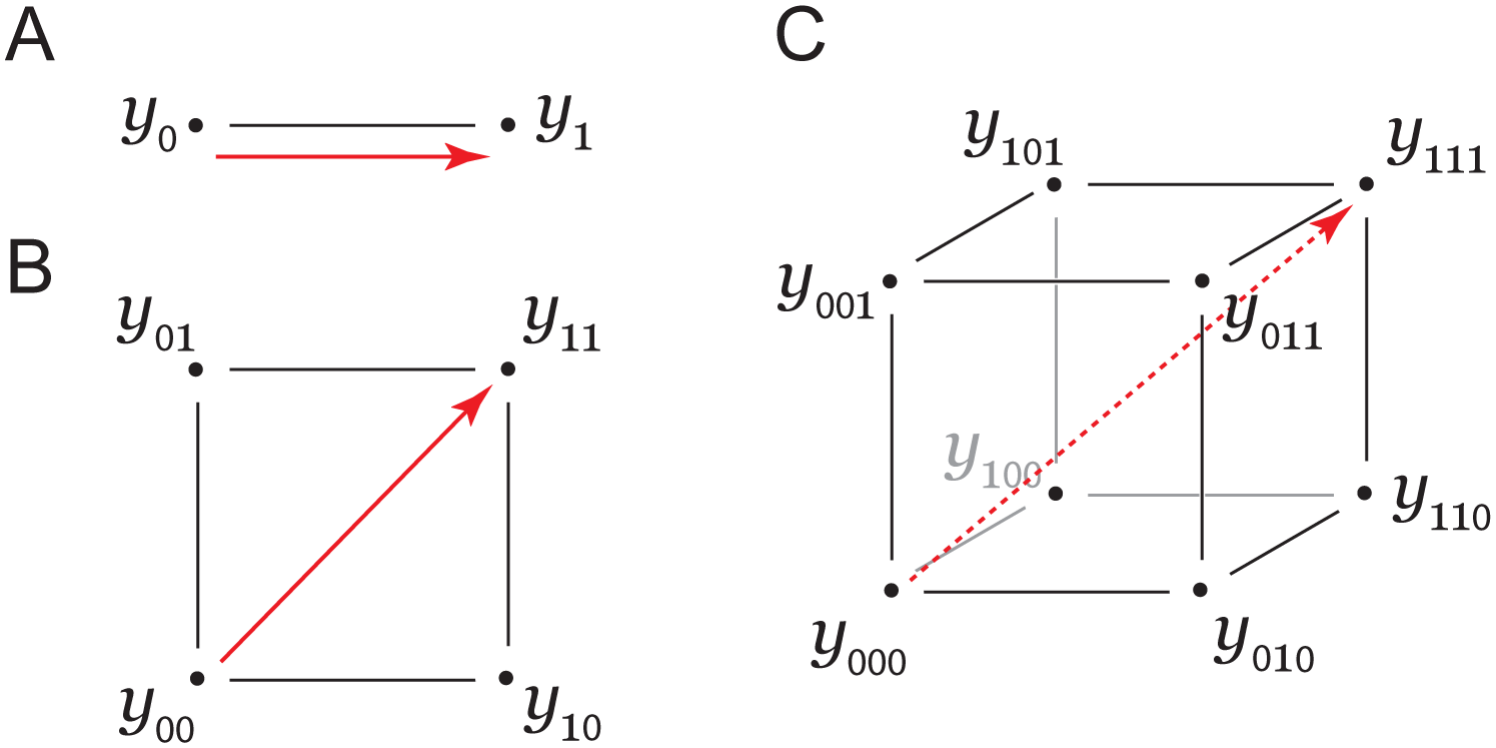
Definitions of genotype, phenotype, and effects of mutations. Representation of (A) single mutant, (B) double mutant, and (C) triple mutant experiments. Phenotypes are denoted by *y*_*g*_, where *g* is the underlying genotype. *g* = {*g*_*N*_,…,*g*_1_} with *g*_*i*_ ∈{0,1}; “0” or “1” indicates the state of the mutable site (e.g., amino acid position). The effect of a single, double, and triple mutation is given by the red arrows. Pairwise (or second-order) epistasis is defined as the differential effect of a mutation depending on the background in which it occurs; for example, in (B) it is the degree to which the effect of one mutation (e.g., *y*_10_−*y*_00_) deviates in the background of the second mutation (*y*_11_−*y*_01_). Thus, the expression for second-order epistasis is (*y*_11_−*y*_10_)−(*y*_01_−*y*_00_). The third order and higher cases are considered in the main text.

We begin with a formal definition of genotype, phenotype, and the representation of mutational effects. Consider a specific sequence comprised of *N* positions as a binary string *g* = {*g*_*N*_,…,*g*_1_} with *g*_*i*_ ∈{0,1}, where “0” and “1” represent the "wild-type" and mutant state of each position, respectively. This defines a total space of 2^*N*^ genotypes. The analysis could be expanded to the case of multiple substitutions per position, but we consider just the binary case for clarity here. Each genotype *g* has an associated phenotype *y*_*g*_, which is of the form that the independent action of two mutations means additivity in *y*. For notational simplicity, we will simply write the genotype in a *k*-bit binary form, where *k* is the order of the mutations that are considered. For example, the effect of a single mutation is simply *y*_1_−*y*_0_, the difference in the phenotype between the mutant and “wild-type” states (Fig 1A). The effect of a double mutant is given by *y*_11_−*y*_00_ (red arrow, Fig 1B), and its linkage through paths of single mutations is defined by a two-dimensional graph (a square network) with four total genotypes. Similarly, a triple mutant effect is *y*_111_−*y*_000_ (red arrow, Fig 1C), and its linkage through paths of single mutations are enumerated on a three-dimensional graph (a cube) with eight total genotypes. More generally, and as described by Horowitz and Fersht [51], the phenotypic effect of any arbitrary *n*-dimensional mutation can be represented by an *n*-dimensional graph with 2^*n*^ total genotypes. Understanding the relationship of the phenotypes of multiple mutants to that of the underlying lower-order mutant states is the essence of epistasis and is described below.

### The biochemical view of epistasis

A well-known approach in biochemistry for analyzing the cooperativity of amino acids in specifying protein structure and function is to use the formalism of thermodynamic mutant cycles [10,51–53], one manifestation of the general principle of epistasis. In this approach, the "phenotype" is typically an equilibrium free energy Δ*G* (e.g., of thermodynamic stability or biochemical activity), and the goal is to obtain information about the structural basis of this phenotype through mutations that represent subtle perturbations of the “wild-type” state. For pairs of mutations, the analysis involves measurements of four variants: “wild-type” ($y_{00}=\Delta G{\;}_{\text{0}}^{\text{o}}$), each single mutant ($y_{01}=\Delta G{\;}_{1}^{\text{o}}$ and $y_{10}=\Delta G{\;}_{2}^{\text{o}}$), and the double mutant ($y_{11}=\Delta G{\;}_{1,2}^{\text{o}}$), where the lower indices designate the mutated positions and the upper index “o” indicates that free energies are relative to the usual biochemical standard state (Fig 1B).

From this, we can compute a coupling free energy between the two mutations (Δ^2^*G*_1,2_) as the degree to which the effect of one mutation (Δ^1^*G*_1_) is different when the same mutation occurs in the background of the other (Δ^1^*G*_1|2_):

$$\begin{array}{l} \Delta^{2\text{​}}G{\;}_{1,2}=\Delta^{\text{​}1\text{​}}G{\;}_{1}{}_{|2}-\Delta^{\text{​}1\text{​}}G{\;}_{1}\\ =(\Delta G{\;}_{1,2}^{\text{o​}}-\Delta G{\;}_{2}^{\text{o​}}\;)-(\Delta G{\;}_{1}^{\text{ o​}}-\Delta G{\;}_{0}^{\text{ o​}}\;) \end{array}$$ (1)

Whereas the Δ*G*^o^ terms are individual measurements and Δ^1^*G* terms are the effects of single mutations relative to “wild-type,” Δ^2^*G* is a second-order epistatic term describing the cooperativity (or nonindependence) of two mutations with respect to the “wild-type” state. This analysis can be expanded to higher order (see [53]). For example, the third-order epistatic term describing the cooperative action of three mutations 1, 2, and 3 (Δ^3^*G*_1,2,3_) is defined as the degree to which the second order epistasis of any two mutations is different in the background of the third mutation:

$$\begin{array}{ll} \Delta^{3\;}G{\;}_{1,2,3} & =\Delta^{\;2\;}G{\;}_{1,2|}{}_{3}\;-\Delta^{\;2\;}G{\;}_{1,2}\\ & =\Delta G{\;}_{1,2,3}^{\text{ o }}\;-\sum_{i<j}^{3}\;\Delta G_{i,j}^{\text{ o }}+\sum_{i}^{3}\;\Delta G_{i}^{\text{ o }}-\Delta G_{0}^{\text{ o }} \end{array}$$ (2)

Note that Δ^3^*G* requires measurement of eight individual genotypes (Fig 1C). More generally, we can define an *n*^th^-order epistatic term (Δ^*n*^*G*), describing the cooperativity of *n* mutations,

$$\begin{array}{ll} \Delta^{\;n\;}G_{1,\ldots ,n} & =\Delta G_{1,\ldots ,n}^{\text{ o}}\;+\;{\left(-1\right)}^{1}\sum_{i_{1}<i_{2}<\ldots <i_{n-1}}^{n}\Delta G_{i_{1},i_{2},\ldots ,i_{n-1}}^{\text{ o}}\\ & +\;{\left(-1\right)}^{2}\sum_{i_{1}<i_{2}<\ldots <i_{n-2}}^{n}\Delta G_{i_{1},i_{2},\ldots ,i_{n-2}}^{\text{ o}}\;+\ldots +\;{\left(-1\right)}^{n}\Delta G_{0}^{\text{ o}} \end{array}$$ (3)

It is possible to write this expansion in a compact matrix form:

$$\bar{\lambda }=G\;\bar{y}$$ (4)

where $\bar{\lambda }$ is the vector of 2^*n*^ epistasis terms of all orders and $\bar{y}$ is the vector of 2^*n*^ free energies corresponding to phenotypes of all the individual variants listed in binary order. To illustrate, for three mutations *n* = 3, we obtain

$$\left(\begin{array}{c} \lambda_{000}\\ \lambda_{001}\\ \lambda_{010}\\ \lambda_{011}\\ \lambda_{100}\\ \lambda_{101}\\ \lambda_{110}\\ \lambda_{111} \end{array}\right)=\left(\begin{array}{rrrrrrrr} 1 & 0 & 0 & 0 & 0 & 0 & 0 & 0\\ -1 & 1 & 0 & 0 & 0 & 0 & 0 & 0\\ -1 & 0 & 1 & 0 & 0 & 0 & 0 & 0\\ 1 & -1 & -1 & 1 & 0 & 0 & 0 & 0\\ -1 & 0 & 0 & 0 & 1 & 0 & 0 & 0\\ 1 & -1 & 0 & 0 & -1 & 1 & 0 & 0\\ 1 & 0 & -1 & 0 & -1 & 0 & 1 & 0\\ -1 & 1 & 1 & -1 & 1 & -1 & -1 & 1 \end{array}\;\right)\;*\left(\begin{array}{l} y_{000}\\ y_{001}\\ y_{010}\\ y_{011}\\ y_{100}\\ y_{101}\\ y_{110}\\ y_{111} \end{array}\right)$$

In this representation, lower indices in $\bar{y}$ represent combinations of mutations (e.g., $y_{011}=\Delta G{\;}_{1,2}^{\text{o}}$, a double mutant) and lower indices in $\bar{\lambda }$ represent epistatic order (e.g., $\lambda_{011}=\Delta^{2}G{\;}_{1,2}$, pairwise epistasis between mutations 1 and 2). Thus, Eqs 1 and 2 correspond to multiplying $\bar{y}$ by the fourth or eighth row of ***G***, respectively, to specify *λ*_011_ and *λ*_111_. Note that $\bar{y}$ and $\bar{\lambda }$ contain precisely the same information re-written in a different form. The matrix ***G*** represents an operator linking these two representations of the mutation data. We will return to the nature of the operation in a later section. We can write a recursive definition for ***G*** that defines the mapping between $\bar{y}$ and $\bar{\lambda }$ for all epistatic orders *n*:

$$G_{n+1}=\left(\begin{array}{r} G_{n}\\ -G_{n} \end{array}\begin{array}{l} 0\\ G_{n} \end{array}\right)\text{ with }G_{0}=1$$ (5)

The inverse mapping is defined by $\bar{y}=G^{-1}\bar{\lambda }$. This relationship gives the effect of any combination of mutants (in $\bar{y}$) as a sum over epistatic terms (in $\bar{\lambda }$). This yields, for example, for the energetic effect of three mutations 1, 2, and 3 ($\Delta G{\;}_{1,2,3}^{\text{o}}\;=y_{111}$):

$$\Delta G{\;}_{1,2,3}^{\text{o}}\;=\Delta^{3\;}G{\;}_{1,2,3}\;+\sum_{i<j}^{3}\;\Delta^{\;2\;}G{\;}_{i,j}\;+\sum_{i}^{3}\;\Delta^{\;1\;}G_{i}+\Delta G{\;}_{0}^{\text{ o }}$$ (6)

Thus, in the most general case, the free energy value of a multiple mutation requires knowledge of the effect of the single mutations and all associated epistatic terms. For the triple mutant, this means the “wild-type” phenotype, the three single mutant effects, the three two-way epistatic interactions, and the single three-way epistatic term. This analysis highlights two important properties of epistasis: (1) the lack of any epistatic interactions between mutations dramatically simplifies the description of multiple mutations to just the sum over the underlying single mutation effects, and (2) the absence of lower-order epistatic interactions (e.g., Δ^2^*G*_*i*,*j*_ = 0) does not imply absence of higher-order epistatic terms.

### The ensemble view of epistasis

In contrast to the biochemical definition, the significance of a mutation (and its epistatic interactions) may also be defined not solely with regard to a single reference state as the "wild-type", but as an average over many possible genotypes. As we show below, such averaging more clearly identifies epistatic units within a protein and, in principle, can separate mutant effects that are idiosyncratic to particular proteins from those that generally hold over the selected ensemble of genotypes. The concept of averaging epistasis over genotypic backgrounds is related to "statistical epistasis" in evolutionary biology, in which the effects of combinations of mutations are averaged over genotypes present in a population [2]. It is also analogous to the idea of the “schema average fitness” in the field of genetic algorithms (GA) [54], but as applied in a biological context (see e.g., [45]).

In its complete form, background-averaged epistasis considers averages over all possible genotypes for the remaining positions in the ensemble. For example, if *n* = 3, the epistasis between two positions 1 and 2 is computed as an average over both states of the third position (ε_*11_, with the averaging denoted by a subscript “*”) (see Fig 1C):

$$\varepsilon_{*11}=\frac{1}{2}\{[(y_{111}-y_{110})-(y_{101}-y_{100})]+[(y_{011}-y_{010})-(y_{001}-y_{000})]\}$$ (7)

Thus for *n* = 3, we can write all epistatic terms:

$$\left(\begin{array}{c} \varepsilon_{***}\\ \varepsilon_{**1}\\ \varepsilon_{*1*}\\ \varepsilon_{*11}\\ \varepsilon_{1**}\\ \varepsilon_{1*1}\\ \varepsilon_{11*}\\ \varepsilon_{111} \end{array}\right)=V*\left(\begin{array}{rrrrrrrr} 1 & 1 & 1 & 1 & 1 & 1 & 1 & 1\\ 1 & -1 & 1 & -1 & 1 & -1 & 1 & -1\\ 1 & 1 & -1 & -1 & 1 & 1 & -1 & -1\\ 1 & -1 & -1 & 1 & 1 & -1 & -1 & 1\\ 1 & 1 & 1 & 1 & -1 & -1 & -1 & -1\\ 1 & -1 & 1 & -1 & -1 & 1 & -1 & 1\\ 1 & 1 & -1 & -1 & -1 & -1 & 1 & 1\\ 1 & -1 & -1 & 1 & -1 & 1 & 1 & -1 \end{array}\right)*\left(\begin{array}{l} y_{000}\\ y_{001}\\ y_{010}\\ y_{011}\\ y_{100}\\ y_{101}\\ y_{110}\\ y_{111} \end{array}\right)$$

where ***V*** is a diagonal weighting matrix to account for averaging over different numbers of terms as a function of the order of epistasis; $v_{ii}=(-{1)}^{q_{i}}{/2}^{n-q_{i}}$, where *q*_*i*_ is the order of the epistatic contribution in row *i*. More generally, for any number of mutations *n*:

$$\bar{\varepsilon }=V\;H\;\bar{y}$$ (8)

where $\bar{y}$ is the same vector of phenotypes of variants as defined above, $\bar{\varepsilon }$ is the vector of background-averaged epistatic terms, and ***H*** is the operator for background-averaged epistasis, defined recursively as

$$H_{n+1}=\left(\begin{array}{rr} H_{n} & H_{n}\\ H_{n} & -H_{n} \end{array}\;\right)\text{ with }H_{0}=1$$ (9)

The recursive definition for the weighting matrix ***V*** is

$$V_{n+1}=\left(\begin{array}{cc} \frac{1}{2}V_{n} & 0\\ 0 & -V_{n} \end{array}\right)\text{ with }V_{0}=1$$ (10)

The matrix ***H*** has special significance; its action mathematically corresponds to a generalized Fourier decomposition [55,56] known as the Walsh-Hadamard transform and, therefore, this operation can also be seen as a spectral analysis of the high-dimensional phenotypic landscape defined by the genotypes studied [47–50]. In this transform, the phenotypic effects of combinations of mutations are represented as sums over averaged epistatic terms. We note that strong parallels exist between the Fourier decomposition of a landscape and ANOVA, a statistical analysis based on partitioning of variance among effects and interactions of different orders (see S5 Text for details).

In summary, the definition of epistasis laid out in this section is a global definition over sequence space, averaging the epistatic effects of mutations over the ensemble of all possible variants. In contrast, the biochemical definition given in the previous section is a local one, treating a particular variant as a reference for determining the epistatic effect of mutations.

### Estimating epistasis with linear regression

A third approach for analyzing epistasis is linear regression. For example, when we have a complete dataset of phenotypes of all 2^*n*^ genotypes, we can use regression to define the extent to which epistasis is captured by only considering terms to some order *r*<*n*. That is, whether terms up to the *r*^th^ order are sufficient for effectively capturing the full complexity of a biological system. The standard form for a linear regression is a set of equations:

$$y_{g}=\beta_{0}+\sum_{i=1}^{n}\beta_{i}g_{i}+\sum_{i<j}^{n}\beta_{ij}g_{i}g_{j}+\sum_{i<j<k}^{n}\beta_{ijk}g_{i}g_{j}g_{k}+\ldots +\in_{g}$$ (11)

for each genotype *g*. The *β* terms denote the regression coefficients corresponding to the (epistatic) effects between subscripted positions and ∈_*g*_ is the residual noise term. In matrix form this can be written as

$$\bar{y}=X\bar{\beta }+\bar{\in }$$ (12)

where ***X*** tabulates which regression coefficients are summed over for genotypes *g*. For *n* = 3, regressing to full order, we can write

$$\left(\begin{array}{l} y_{000}\\ y_{001}\\ y_{010}\\ y_{011}\\ y_{100}\\ y_{101}\\ y_{110}\\ y_{111} \end{array}\right)=\left(\begin{array}{cccccccc} 1 & 0 & 0 & 0 & 0 & 0 & 0 & 0\\ 1 & 1 & 0 & 0 & 0 & 0 & 0 & 0\\ 1 & 0 & 1 & 0 & 0 & 0 & 0 & 0\\ 1 & 1 & 1 & 1 & 0 & 0 & 0 & 0\\ 1 & 0 & 0 & 0 & 1 & 0 & 0 & 0\\ 1 & 1 & 0 & 0 & 1 & 1 & 0 & 0\\ 1 & 0 & 1 & 0 & 1 & 0 & 1 & 0\\ 1 & 1 & 1 & 1 & 1 & 1 & 1 & 1 \end{array}\right)*\left(\begin{array}{c} \beta_{000}\\ \beta_{001}\\ \beta_{010}\\ \beta_{011}\\ \beta_{100}\\ \beta_{101}\\ \beta_{110}\\ \beta_{111} \end{array}\right)+\bar{\in }$$

following the same rule for the lower indices as before. ***X*** has the recursive definition:

$$X_{n+1}=\left(\begin{array}{ll} X_{n} & 0\\ X_{n} & X_{n} \end{array}\right)\text{ with }X_{0}=1$$ (13)

It is worth noting that the inverse of ***X*** is ***X***^−1^ = ***G***, the operator for biochemical epistasis (Eq 5; see also S1 Text). Thus, the multidimensional mutant-cycle analysis is indistinguishable from regression to full order (*r* = *n*), which is an exact mapping without residual noise ($\bar{\in }\;=0$).

However, the usual aim of regression is to approximate the data with fewer coefficients than there are data points, i.e., *r*<*n*. To express this, we simply remove the columns from ***X*** that refer to the epistatic orders excluded from the regression (i.e., >*r*): ***X*** is multiplied by a 2^*n*^ -by-*m* matrix ***Q***, the identity matrix with columns corresponding to epistatic orders higher than *r* removed. *m* is the number of epistatic terms up to *r* and is given by $m=\sum_{i=0}^{r}\left({}_{i}^{n}\right)$. Thus for regression to order *r*, we can define $\hat{X}=XQ$, and write

$$\bar{y}=\hat{X}\hat{\beta }+\hat{\in }$$ (14)

The linear regression is performed by solving the so-called normal equations

$$\hat{\beta }=({\hat{X}}^{T}\hat{X}{)}^{-1}{\hat{X}}^{T}\bar{y}$$ (15)

where ${\hat{X}}^{T}$ is the transpose of $\hat{X}$. The product ${\hat{X}}^{T}\hat{X}$ is necessarily square and invertible as long as $\hat{X}$ is full column rank and hence ${\hat{X}}^{T}\hat{X}$ is full rank. Note that in this analysis we compute epistatic terms only up to the *r*^th^ order, but use phenotype/fitness data of all 2^*n*^ combinations of mutants. The more general case, in which we estimate epistatic terms with less than 2^*n*^ data points, is distinct and is discussed below.

If the biochemical definition of epistasis is a local one, exploring the coupling of mutations of all order with regard to one "wild-type" reference, and the ensemble view of epistasis is a global one, assessing the coupling of mutations of all order averaged over all possible genotypes, then the regression view of epistasis is an attempt to project to a lower dimension—capturing epistasis as much as possible with low-order terms.

### Link between the formalisms

The analysis presented above leads to a simple unifying concept underlying the calculations of epistasis. In general, all the calculations are a mapping from the space of phenotypic measurements of genotypes $\bar{y}$ to epistatic coefficients $\bar{\omega }$ in a general form $\bar{\omega }=\Omega {\;}_{\text{epi}}\;\bar{y}$, where Ω_epi_ is the epistasis operator. We give the bottom line of the different operators below; their formal mathematical derivations can be found in S1 Text.

The most general situation is that of the background-averaged epistasis with averaging over the complete space of possible genotypes. In this case

$$\Omega {\;}_{\text{epi}}=V\;H,$$ (16)

where ***H*** is a 2^*n*^×2^*n*^ matrix corresponding to the Walsh-Hadamard transform (*n* is the number of mutated sites) and ***V*** is a matrix of weights to normalize for the different numbers of terms for epistasis of different orders. The biochemical definition of epistasis using one "wild-type" sequence as a reference is a sub-sampling of terms in the Hadamard transform. In this case

$$\Omega {\;}_{\text{epi}}=V\;X^{T}\;H,$$ (17)

where ***X*** is as defined in Eq 13. In essence, ***X***^*T*^ picks out the terms in ***H*** that concern the “wild-type” background. Note that both these mappings are one-to-one, such that the number of epistatic terms (in $\bar{\omega }$) is equal to the number of phenotypic measurements (in $\bar{y}$) and no information is lost. In contrast, regression to lower orders necessarily implies fewer epistatic terms than data points, which means the mapping is compressive and information is lost. In this case

$$\Omega {\;}_{\text{epi}}=V\;X^{T}S\;H,$$ (18)

where ***S*** (≡***QQ***^*T*^) is the identity matrix but with zeros on the diagonal at the orders that are higher than those over which we regress. From a computational point of view, it is interesting to note that regression using the Hadamard transform makes matrix inversion unnecessary (compare with Eq 15).

The fundamental point is that all three formalisms for computing epistasis are just versions of the Walsh-Hadamard transform, with weights selected as appropriate for the choice of a single reference sequence or restrictions on the order of epistatic terms considered. The mathematical underpinnings of these relationships have been previously noted and explained [45,47–50], though the connections to experimental studies in biochemistry and evolutionary biology have been incomplete and underappreciated by the broader scientific community. For example, ensemble and biochemical views of epistasis correspond to Fourier and Taylor expansions, respectively, of multi-dimensional landscapes [47]. The former captures global landscape properties and the latter evaluates the local structure around a particular genotype. Interestingly, the two representations are also mathematically interchangeable (up to weighting factors) by simply changing the representation of genotypes from *g*_*i*_ ∈{0,1} to *σ*_*i*_ ∈{−1,1} in an expansion of the form of the regression equation (see Eq 11 and S4 Text). Understanding the connection between the mathematical descriptions and experimental studies of phenotype landscapes as practiced in different fields is important in guiding future work.

### Empirical examples

**Fig 2:**
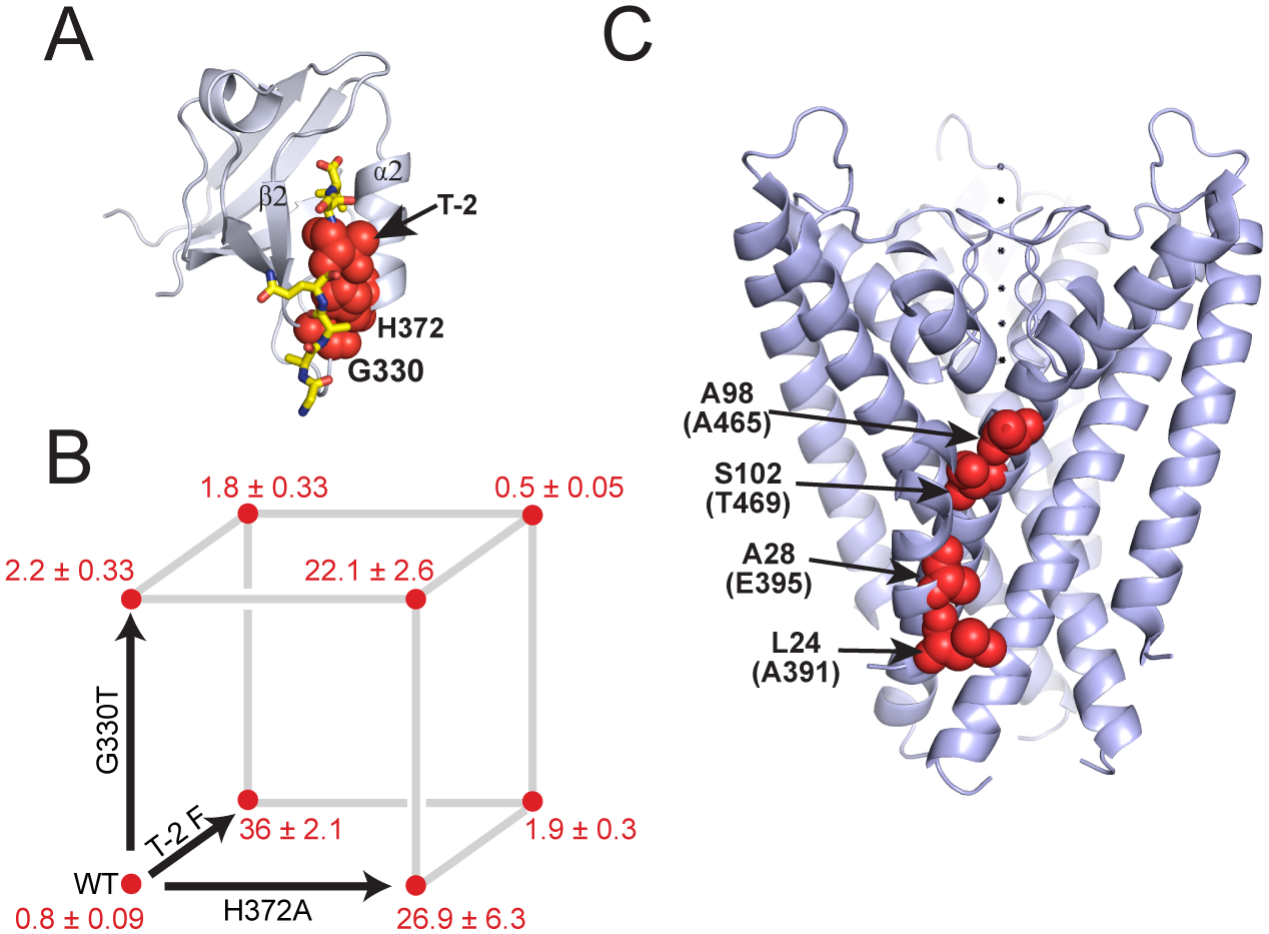
Examples of epistasis in a PDZ domain (A) and a K+ ion channel (B). (**A)** PDZ domains are small, mixed *αβ* proteins that bind target peptide ligand (in yellow stick bonds) in a groove formed between the *β*2 and *α*2 elements (PSD95^pdz3^ shown, Protein Data Bank (PDB) accession 1BE9). The study discussed in the main text and in Table 1 is focused on the epistatic interactions between three amino acid positions—two in the PDZ domain (H372 and G330) and one in the ligand (T-2) (red spheres). (**B)** a thermodynamic cube representing the energetics of mutations at the three positions; values are equilibrium dissociation constants (*K*_*d*_) for the target ligand (CRIPT [58]) in *μ*M for all eight possible combination of mutations; errors represent standard deviation. (**C)** structure of the homotetrameric KcsA K^+^ ion channel (PDB accession 1K4C), showing the four positions selected for mutation in Sadovsky and Yifrach (in red spheres, shown only for one subunit for clarity) [60]. Note that the experiments were carried out in the Shaker K^+^ ion channel, and the positions in Shaker numbering are given in parentheses. The positions form a network that roughly links the intracellular activation gate and the selectivity filter.

To illustrate the different analyses of epistasis, we begin with a small case study of three spatially proximal mutations that define a switch in ligand specificity in PSD95^pdz3^, a member of the PDZ family of protein interaction modules (Fig 2A). Two mutations are located in the PDZ3 domain itself (G330T and H372A) and one mutation is in its cognate ligand peptide (T-2F). The phenotype is the binding affinity, *K*_*d*_, and the absence of epistasis implies additivity in the corresponding free energy, expressed as Δ*G*^o^ = *RT*ln*K*_*d*_. (Binding affinities for this system are measured in [57] and given in Fig 2B) These quantitative phenotypes are then transformed into epistatic terms using Eqs 16–18 (Table 1).

**Table 1:** Interaction terms after applying the three different transforms to the PDZ–ligand dataset with three mutable positions: three-way mutant cycle, background-averaged epistasis, and regression (to second order).

Genotype^1^	Free Energy^2^	Interaction Terms^3^	Mutant Cycle	Background-Averaged Epistasis	Regression Terms
THG	$\bar{y}$		$\bar{\lambda }$	$\bar{\varepsilon }$	$\bar{\beta }$
000	-8.17 (0.07)	***	-8.17 (0.07)	-7.24 (0.03)	-7.96 (0.06)
001	-7.58 (0.09)	**1	0.59 (0.11)	-0.51 (0.06)	0.17 (0.10)
010	-6.13 (0.14)	*1*	2.05 (0.15)	0.23 (0.06)	1.63 (0.13)
011	-6.24 (0.07)	*11	-0.70 (0.19)	0.13 (0.12)	0.13 (0.12)
100	-5.96 (0.03)	1**	2.22 (0.07)	-0.41 (0.06)	1.80 (0.08)
101	-7.70 (0.11)	1*1	-2.33 (0.16)	-1.50 (0.12)	-1.50 (0.12)
110	-7.67 (0.09)	11*	-3.76 (0.18)	-2.92 (0.12)	-2.92 (0.12)
111	-8.45 (0.06)	111	1.67 (0.25)	1.67 (0.25)	0 (0.00)

^1^ The three mutations are T-2F in the ligand and H372A and G330T in the protein, respectively. They are designated in this column as “THG.”

^2^ Free energies are in kcal/mol, with standard deviation in parentheses.

^3^ Interacting positions are in the same order as genotypes, e.g., “*11” indicates the epistasis between amino acid positions 372 and 330 in PSD95-PDZ3.

Standard deviations in epistatic terms are given in parentheses and calculated according to $\bar{\delta \omega }=(\Omega {\;}_{\text{epi}}\;\circ \Omega {\;}_{\text{epi}}\;\bar{\delta y}\circ \bar{\delta y}{)}^{1/2}$, where *δ*s designate the error vectors and ∘ stands for the element-wise product (see also S2 Text).

A number of simple mathematical relationships are evident in the data. First, regression is carried out only to the second order, and therefore the third-order epistatic term for this analysis does not exist (or, equivalently, is set to zero if the epistatic vector $\hat{\beta }$ is defined to be of full length 2^*n*^). Second, some numerical equalities exist. The regression terms at the highest order (second, in this case) are equal to the corresponding terms for the averaged epistasis. This is because ***X***^*T*^***S*** sets columns representing orders higher than the regression order to zero, leaving rows corresponding to the highest regression order with only one non-zero element on the diagonal. For these rows, the entries in the epistasis operators ***V X***^*T*^***S H*** and ***V H*** are equal. Another more trivial equality is the highest-order term for the mutant-cycle and averaged epistasis formalisms; there is only one contribution for the highest order and, therefore, no backgrounds over which to average.

The data also illustrate the key properties of the different formalisms. The G330T, H372A, and T-2F mutations represent a collectively cooperative set of perturbations, as indicated by a significant third-order epistatic term by both mutant cycle and background-averaged definitions (*λ*_111_ = *ε*_111_ = 1.67 kcal mol^−1^). But the three formalisms differ in the energetic value of the lower-order epistatic terms. For example, G330T is essentially neutral for “wild-type” ligand binding but shows a dramatic gain in affinity in the context of the T-2F ligand; thus, a large second-order epistatic term by the biochemical definition (*λ*_101_ = −2.33 kcal mol^−1^). However, the coupling between G330T and T-2F is nearly negligible in the background of H372A; as a consequence, the background-averaged second-order epistasis term *ε*_1*1_ is smaller (−1.5 kcal mol^−1^). Similarly, both biochemical and regression formalisms assign a large first-order effect to the T-2F (1**) and H372A (*1*) single mutations, while the corresponding background-averaged terms are nearly insignificant. For example, the free energy effect of mutating the ligand (T-2F, *λ*_010_) is 2.22 kcal mol^−1^ in the “wild-type” background, but is −1.54 kcal mol^−1^ in the background of the H372A mutation—a nearly complete reversal of the effect of this mutation depending on context. Thus, with background averaging, the first-order term for T-2F (*ε*_1**_) is close to zero. This makes sense given the experiment described in Fig 2, and, more broadly, given the known specificities in the PDZ family [59], the mutation should not be thought of as a general determinant of ligand affinity. T-2F may have a disrupting effect on the function from the perspective of a specific PDZ domain (the “wild-type”), but from the perspective of the protein family (in which various different functional domain–ligand combinations are found) a phenylalanine at position -2 in the ligand is not necessarily detrimental to binding affinity. Instead, it is a conditional determinant with an effect that depends on the identity of the proximal amino acid in the PDZ domain.

The analysis of other combinatorial mutation datasets reinforces these conclusions. For example, high-quality measurements comprising a fourth-order thermodynamic analysis of epistasis is available for the Shaker potassium channel (data from [60] and [61], Fig 2C, Table 2), where the phenotype observed is the activation free energy for opening of the ion channel pore [61]. Using the standard biochemical formalism for epistasis, the work of Sadavosky and Yifrach [60] demonstrates large high-order epistasis between four mutations at sites forming a path between the intracellular crossing of transmembrane helices (the so-called “activation gate”) and the selectivity filter for ions (Fig 2C, [61]). The biologically interesting finding is that for this system of mutations, the magnitude of epistasis rises with increasing order of the epistasis; that is, Δ^4^*G*> Δ^3^*G>* Δ^2^*G>* Δ^1^*G* (Table 3, $|\bar{\lambda }{|}_{\text{mean}}$), a result that suggests the collective action of this systems of residues with regard to pore opening. We compared the biochemical and background-averaged epistasis for this system of four mutations (Fig 2C, Table 2, and complete analysis in S3 Text). The analysis shows that the background-averaged epistasis enhances the essential point of Sadovsky and Yifrach; the fourth-order epistatic term dominates (Table 3, $|\bar{\varepsilon }{|}_{\text{mean}}$), and all lower terms are weak. As in the case of the PDZ domain, the reason for this is that the lower-order epistatic effects are conditional on the background of other mutations and are correspondingly assigned less significance. This analysis clarifies the notion that this system of residues comprises a collectively acting, cooperative network underlying channel gating.

**Table 2:** Interaction terms based on the standard mutant cycle formalism ($\bar{\lambda }$) and on background-averaged epistasis ($\bar{\varepsilon }$) for pore-opening free energies in the Shaker K^+^ voltage-gated channel. As for the PDZ domain (Table 1), background averaging modulates epistasis at each level given the existence of higher-order terms. Primary data are from [60] and [61].

Genotype^1^	Δ*G*_open_^2^	Interaction Terms	Mutant Cycle^2^	Background-Averaged Epistasis^2^
	$\bar{y}$		$\bar{\lambda }$	$\bar{\varepsilon }$
0000	-1.97 (0.05)	****	-1.97 (0.05)	-8.33 (0.05)
0001	-7.05 (0.12)	***1	-5.08 (0.13)	-0.64 (0.10)
0010	-13.57 (0.29)	**1*	-11.60 (0.29)	-3.52 (0.10)
0011	-9.47 (0.25)	**11	9.18 (0.40)	2.97 (0.20)
0100	-7.97 (0.34)	*1**	-6.00 (0.34)	-1.09 (0.10)
0101	-8.11 (0.19)	*1*1	4.94 (0.41)	-1.13 (0.20)
0110	-10.01 (0.33)	*11*	9.56 (0.56)	1.46 (0.20)
0111	-13.50 (0.32)	*111	-12.53 (0.73)	-3.00 (0.40)
1000	-7.04 (0.21)	1***	-5.07 (0.22)	1.25 (0.10)
1001	-6.58 (0.08)	1**1	5.54 (0.26)	1.02 (0.20)
1010	-8.42 (0.13)	1*1*	10.22 (0.38)	3.68 (0.20)
1011	-8.20 (0.16)	1*11	-9.42 (0.51)	0.12 (0.40)
1100	-5.05 (0.12)	11**	7.99 (0.42)	1.58 (0.20)
1101	-8.80 (0.09)	11*1	-9.15 (0.49)	0.39 (0.40)
1110	-10.07 (0.11)	111*	-13.20 (0.63)	-3.67 (0.40)
1111	-7.52 (0.04)	1111	19.07 (0.81)	19.07 (0.81)

^1^ The four mutations are T469A, A465V, E395A, and A391V (corresponding to the bits in the first column in left-to-right order).

^2^ Standard deviations of epistatic terms are given in parentheses and computed according to $\bar{\delta \omega }=(\Omega {\;}_{\text{epi}}\circ \Omega {\;}_{\text{epi}}\;\bar{\delta y}\circ \bar{\delta y}{)}^{1/2}$ (see S2 Text).

**Table 3:** Mean absolute values of interaction terms for the four-mutation network in the Shaker K^+^ channel. This analysis recapitulates the basic finding of Sadovsky and Yifrach [60] that these positions comprise a cooperative unit, a result that is further clarified with background averaging.

Epistatic Order^1^	Mutant Cycle^2^	Background-Averaged Epistasis^2^
	$|\bar{\lambda }{|}_{\text{ mean}}$	$|\bar{\varepsilon }{|}_{\text{ mean}}$
0	1.97 (0.05)	8.33 (0.05)
1	6.94 (0.26)	1.63 (0.10)
2	7.91 (0.42)	1.98 (0.20)
3	11.08 (0.60)	1.79 (0.40)
4	19.07 (0.81)	19.07 (0.81)

^1^ Order over which the absolute values of epistatic terms are averaged.

^2^ Errors on the mean are given in parentheses.

These examples show that background averaging has the effect of “correcting” mutational effects for the existence of higher-order epistatic interactions. Without background averaging, the effect of a mutation (at any order) idiosyncratically depends on a particular reference genotype and will fail to account for higher-order epistasis that modulates the observed mutational effect. Thus, background averaging provides a measure of the effects of mutation that represents its general value over many related systems and, more appropriately, represents the cooperative unit within which the mutation operates. Note that the degree of averaging depends on the number of mutated sites and, thus, the interpretation of mutational effects will depend on the scale of the experimental study. As we will discuss in the next section, finding good averaging ensembles is crucial for background-averaged epistasis to be a useful quantity. This is not only in terms of elucidating general physical mechanisms at play in the system but also for being able to accurately predict the effects of mutations in an individual system.

### The epistatic structure of larger systems

The analytical expressions in Eqs 16–18 involve the measurement of phenotypes ($\bar{y}$) for all 2^*n*^ combinatorial mutants, a fact that exposes two fundamental problems. First, it is only practical when *n* is small. In such cases (e.g., Fig 2, *n* = 3 or 4), the data can be combinatorially complete, permitting a full analysis—the local and global structure of epistasis, possible evolutionary trajectories, and adaptive trade-offs [62,63]. But for the typical size of protein domains (*n*∼150), the combinatorial complexity of mutations precludes the collection of complete datasets. Second, even if it were possible, the sampling of all genotypes is not desired; indeed, the majority of systems in such an ensemble are unlikely to be functional, and averages over them are not meaningful with regard to learning the epistatic structure of native systems. How then can we apply these epistasis formalisms in practice, especially with regard to background averaging?

To develop general principles, we begin with two obvious approaches that lead to well-defined alternative expressions for averaged epistasis. First, consider the case in which the data are only "locally complete;" that is, we have all possible mutants up to a certain order *p*≤*n*. We can then define a measure that is intermediate between epistasis with a single reference genotype and epistasis with full background averaging, which we will refer to as the *partial* background-averaged epistasis. For example, for three positions (*n* = 3) with data complete only up to order (*p* = 2), the partial background-averaged effect of the first position (rightmost lower index) is calculated as ε_**1,*p*_ = (*y*_001_−*y*_000_+*y*_011_−*y*_010+_y_101_−*y*_100_)/3. Compared to the full background-averaged epistasis, the partial averages just leave out the last term, *y*_111_−*y*_110_, which represents the unavailable phenotype of the triple mutant *y*_111_. More generally, we can define this measure of epistasis as another special case of the Hadamard transform:

$${\bar{\varepsilon }}_{p}=W_{{}_{\;p}}\left(Z_{{}_{p}}\;\circ H\right)\bar{y},$$ (19)

where ∘ designates the element-wise product. ***W***_*p*_ is again a diagonal weighting vector, now given by $w_{ii}=(-{1)}^{q_{i}}/T_{p,q_{i}}$, where *q*_*i*_ is the epistatic order associated with row *i*, as defined earlier, and $T_{p,q_{i}}=\sum_{j=0}^{p-q_{i}}\left({}_{j}^{n-q_{i}}\right)$. Note that *p*≥*q*_*i*_ because mutants of order higher than *p* are considered absent in the dataset.

**Fig 3:**
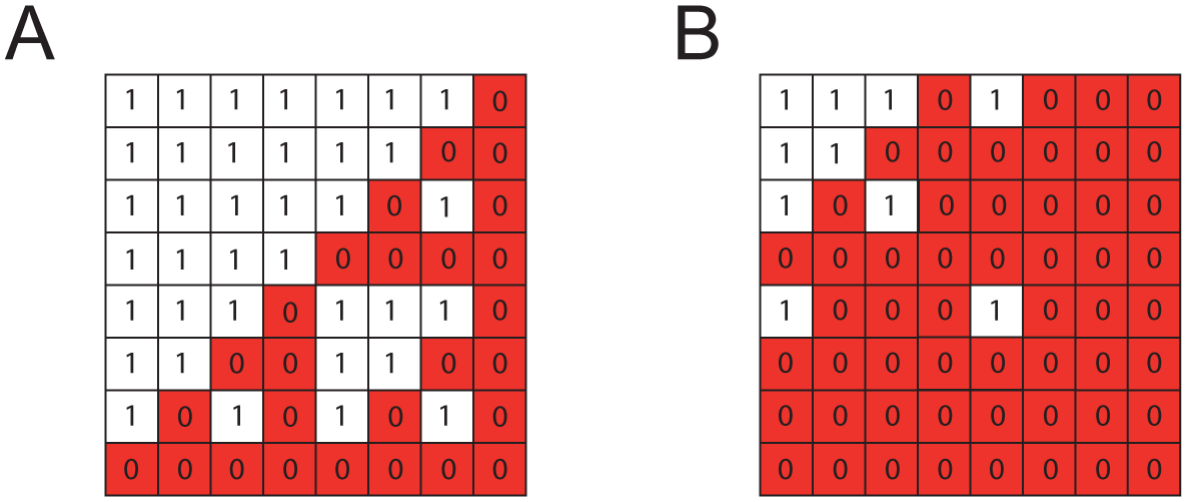
Examples of matrices *Z*_*p*_ introduced to calculate the partial background-averaged epistasis for *n* = 3. (A) ***Z***_2_ for when data for mutants up to second-order is available and (B) ***Z***_1_ for when only first-order mutants are available. Both matrices are self-similar, which allows their generation for arbitrary order, and are related to the logic Sierpinski triangle. For example, ***Z***_2_ = 1−***A***Σ, where ***A*** is the anti-diagonal identity matrix and Σ is the Sierpinski matrix (i.e., multigrade AND in Boolean logic) for three inputs.

The matrix ***Z***_*p*_ simply serves to multiply by zero the terms in the Hadamard matrix that include orders higher than *p*. Interestingly, the ***Z***_*p*_ matrices display a self-similar hierarchical pattern (Fig 3) and are related to Sierpinski triangles (see [64]). This permits a recursive definition in both *n* and *p* for the product ***Z***_*p*_∘***H***, which we will designate as ***F***_*n*,*p*_:

$$F_{n,p}=\left(\begin{array}{ll} F_{{\;}_{n-1,p}} & F_{{\;}_{n-1,p-1}}\\ F_{{\;}_{n-1,p-1}} & -F_{{\;}_{n-1,p-1}} \end{array}\right)\;$$ (20)

with ***F***_*n*,*p*_ = ***H***_*n*_ for *n*≤*p*, and ***F***_*n*,0_ is a 2^*n*^×2^*n*^ matrix of zeros, except for a 1 in the upper left corner. This analysis assumes that data are complete up to order *p*. If not, analytical schemes for background-averaged epistasis such as Eqs 19 and 20 are not obvious.

A second analytically tractable case for incomplete data arises in regression, in which the idea is to estimate epistatic terms up to a specified order from available data. This involves solving a set of equations similar to the normal equations:

$$\tilde{\beta }=Q\;{\left({\tilde{X}}^{T}\;\tilde{X}\right)}^{-1}{\tilde{X}}^{T}M\;\bar{y}$$ (21)

where ***M*** is an *s*×2^*n*^ matrix constructed from the 2^*n*^ by 2^*n*^ identity matrix by deleting the 2^*n*^−*s* rows corresponding to the unavailable phenotypic data, and $\tilde{X}=M\;X\;Q$, with ***Q*** defined as above. In order for this system of equations to be solvable, a necessary constraint is that *s*≥*m*; that is, the number of data points available should be larger than or equal to the number of regression parameters. In addition, the data must be such that it is possible to uniquely solve for all epistatic terms in the regression. For example, if two mutations always co-occur in the data, it is obviously impossible to calculate their independent effects. In such cases, the number of solutions to Eq 21 is infinite (${\tilde{X}}^{T}\;\tilde{X}$ is not invertible).

In practice, even with "high-throughput" assays, we can only hope to measure a tiny fraction of all combinatorial mutants due to the vast number of possibilities. In this situation, the problem of inferring epistasis by regression may be further constrained by imposing additional conditions, termed regularization. For example, kernel ridge regression [65] and least absolute shrinkage and selection operator (LASSO) [66] include a weighted norm of the regression coefficients in the minimization procedure. Regularization comes with its own set of caveats [67], but its application is, unlike the approaches in Eqs 19 and 21, not conditional on specific structure of the data or depth of coverage.

However, none of these approaches directly address the problem of optimally defining appropriate ensembles of genotypes over which averages should be taken. In principle, the idea should be to perform background averaging over a representative ensemble of systems that show invariance of functional properties of interest. How can we generally find such ensembles without the impractical notion of exhaustive functional analysis of the space of possible genotypes? One idea is motivated by the empirical finding of sparsity in the pattern of key epistatic interactions within biological systems. Indeed, evidence suggests that, in proteins, the architecture is to have a small subset of amino acids that shows strong and distributed epistatic couplings surrounded by a majority of amino acids that are more weakly and locally coupled [60,68–71]. Thus, protein sequences can show extraordinary divergence while preserving folding and function, and only a small set of epistatic constraints can suffice to computationally build synthetic proteins that recapitulate these properties [72,73]. More generally, the notion of a sparse core of strong couplings surrounded by a milieu of weak couplings has been argued to be a signature of evolvable systems [74]. If it can be more generally verified, the notion of sparsity can be exploited to define relevant strategies for optimally learning the epistatic structure of natural systems. For example, one approach is to minimize the so-called $l_{1}$ -norm (the sum of absolute values of the epistatic coefficients [66]) in a constrained optimization, while projecting onto background-averaged epistatic terms:

$$\underset{\bar{\varepsilon }}{\text{min}}||\bar{\varepsilon }{\left|\right|}_{1}\text{ subject to }\bar{y}=H^{-1}\;V^{-1}\;\bar{\varepsilon }$$ (22)

This procedure is akin to the technique of compressive sensing [75,76], a powerful approach used in signal processing to recognize the low-dimensional space in which the relevant features of a high-dimensional dataset occur given sparsity of these features. The application of this theory for mapping biological epistasis has, to our knowledge, not been reported before, but its value might be explored with focused high-order mutational analyses in specific well-chosen model systems. This has the potential to link the study of epistasis to a formal theory of signal reconstruction [75,76], which may help define optimal strategies for data collection. The necessary technologies for developing these ideas are now becoming available.

It is worth pointing out that a class of approaches that use ensemble-averaged information to understand complex biological systems has been developed and experimentally tested. Statistical methods that operate on multiple sequence alignments [71,77–82] calculate quantities that estimate the coevolution of amino acids in the sampling of sequences comprising the alignment. In this regard, coevolution can be seen as a form of background-averaged pairwise epistasis in which the ensemble of genotypes for averaging is defined by homology. Importantly, these approaches have been successful at revealing a hierarchy of cooperative interactions between amino acids that range from local structural contacts in protein tertiary structures [81–83] to more global functional modes [71,84,85]. Coevolution only provides averaged pairwise epistatic terms, but studies show that it is possible to use this information to computationally design artificial sequences that fold and biochemically function in a manner similar to their natural counterparts [72,73]. Thus, for defining good experimental approaches to elucidating epistatic structures, a conceptual advance may come from formally mapping the constrained optimization problem described in Eq 22 to the kind of ensemble averaging that underlies the statistical coevolution approaches.

## Discussion

A fundamental problem is to define the epistatic structure of biological systems, which holds the key to understanding how phenotype arises from genotype. Here we describe a unified mathematical foundation for epistasis in which different approaches are versions of a single mathematical formalism—the weighted Walsh-Hadamard transform. In the most general case, this transform corresponds to an averaging of mutant effects over all possible genetic backgrounds at every order of epistasis. This approach corrects the effect of mutations at every level of epistasis for higher order terms. Importantly, it represents the degree to which the effects of mutations are transferable from one model system to another—the usual purpose of most mutagenesis studies. In contrast, the thermodynamic mutant cycle (commonly used in biochemistry) [51] constitutes a special case of taking a single reference genotype and thus no averaging [60,61,86–90]. This analysis represents the effects of mutations that are specific to a particular model system. Regression (commonly used in evolutionary biology) is an attempt to capture features of a system with epistatic terms up to a defined lower order, often to bound the extent of epistasis or to predict the effects of combinations of mutations [33,91]. The similarity of the regression operator to that of the mutant cycle (see Eq 13) indicates that this approach is also focused around the local mutational environment of a chosen reference sequence.

Overall, background averaging would seem to provide the most informative representation of the general effect of a mutation. However, with the exception of very small-scale studies focused on the local mutational environment of extant systems, it is both impractical and logically flawed to collect combinatorially complete mutation datasets for any system. Thus, the essence of the problem is to define optimal strategies for collecting data on ensembles of genotypes that is sufficient for discovering the biologically relevant epistatic structure of systems.

The notion of sparsity of epistatic interactions provides a general basis for developing such a strategy, and it will be interesting to test practical applications of this concept (e.g., Eq 22) in future work. Defining optimal data collection strategies will not only provide practical tools to probe specific systems but also might guide us to principles underlying the "design" of these systems through the process of evolution and help the rational design of new systems. The mathematical relations discussed here provide a foundation to advance such understanding.

## Supporting Information

S1 Text Additional proofs; expressing epistasis operators as Hadamard transforms. (PDF)

S2 Text Error propagation in biochemical and background-averaged epistasis. (PDF)

S3 Text Analysis of epistasis in a K+ channel. (PDF)

S4 Text Fourier and Taylor decompositions as a result of genotype definition. (PDF)

S5 Text Relation between Fourier decomposition and ANOVA. (PDF)

S1 FigPropagation of errors in epistatic terms due to noise in the measured data.Plotted in the main graph are the widths (SD) of the histograms of epistatic terms of each order for a simulated flat dataset with *N* = 14 and a fixed Gaussian noise with σ = 1, for both biochemical and background-averaged epistasis. The inset on the right is an example of the histogram for the calculated 7^th^ order background-averaged contributions. Straight lines in the main graph have the appropriate slopes to indicate an increase in uncertainty by a factor 2 (lower line) or a factor $\sqrt{2}$ (upper line) per order, respectively, and intersect at *N* = 14, in accordance with the expectations of propagation of errors. The intercept of the fit of the biochemical epistasis with the y-axis corresponds to the standard deviation of the noise of the dataset σ = 1.(PDF)

## References

[1] Wells JA (1990) Additivity of mutational effects in proteins. *Biochemistry* 29:8509 2271534 10.1021/bi00489a001

[2] Phillips PC (2008) Epistasis–the essential role of gene interactions in the structure and evolution of genetic systems. *Nat Rev Genet* 9:855 10.1038/nrg2452 18852697 PMC2689140

[3] Costanzo M, Baryshnikova A, Myers CL, Andrews B, Boone C (2011) Charting the genetic interaction map of a cell. *Curr Opin Biotechnol* 22:66 10.1016/j.copbio.2010.11.001 21111604

[4] Lehner B (2011) Molecular mechanisms of epistasis within and between genes. *Trends Genet* 27:323 10.1016/j.tig.2011.05.007 21684621

[5] Dowell RD, Ryan O, Jansen A, Cheung D, Agarwala S, et al (2010) Genotype to phenotype: a complex problem. *Science* 328:469 10.1126/science.1189015 20413493 PMC4412269

[6] Lunzer M, Golding GB, Dean AM (2010) Pervasive cryptic epistasis in molecular evolution. *PLoS Genet* 6:e1001162 10.1371/journal.pgen.1001162 20975933 PMC2958800

[7] Kryazhimskiy S, Dushoff J, Bazykin GA, Plotkin JB (2011) Prevalence of epistasis in the evolution of influenza A surface proteins. *PLoS Genet* 7:e1001301 10.1371/journal.pgen.1001301 21390205 PMC3040651

[8] Bateson W (1908) Facts limiting the theory of heredity. *Science* 26:647. 10.1126/science.26.672.649 17796786

[9] Fisher RA (1918) The correlation between relatives on the supposition of Mendelian inheritance. *Trans R Soc Edinb* 52:399.

[10] Horovitz A (1987) Non-additivity in protein-protein interactions. *J Mol Biol* 196:733 3681975 10.1016/0022-2836(87)90045-3

[11] Cordes MH, Davidson AR, Sauer RT (1996) Sequence space, folding and protein design. *Curr Opin Struct Biol* 6:3 8696970 10.1016/s0959-440x(96)80088-1

[12] Horovitz A, Bochkareva ES, Yifrach O, Girshovich AS (1994) Prediction of an inter-residue interaction in the chaperonin GroEL from multiple sequence alignment is confirmed by double-mutant-cycle analysis. *J Mol Biol* 238:133 7908986 10.1006/jmbi.1994.1275

[13] Dill KA (1997) Additivity principles in biochemistry. *J Biol Chem* 272:701 8995351 10.1074/jbc.272.2.701

[14] Jain RK, Ranganathan R (2004) Local complexity of amino acid interactions in a protein core. *Proc Natl Acad Sci USA* 101:111 14684834 10.1073/pnas.2534352100 PMC314147

[15] Lander ES, Schork NJ (1994) Genetic dissection of complex traits. *Science* 265:2037 8091226 10.1126/science.8091226

[16] Pettersson M, Besnier F, Siegel PB, Carlborg O (2011) Replication and explorations of high-order epistasis using a large advanced intercross line pedigree. *PLoS Genet* 7:e1002180 10.1371/journal.pgen.1002180 21814519 PMC3140984

[17] Kouyos RD, Leventhal GE, Hinkley T, Haddad M, Whitcomb JM, et al (2012) Exploring the complexity of the HIV-1 fitness landscape. *PLoS Genet* 8:e1002551 10.1371/journal.pgen.1002551 22412384 PMC3297571

[18] Brem RB, Kruglyak L (2005) The landscape of genetic complexity across 5,700 gene expression traits in yeast. *Proc Natl Acad Sci USA* 102:1572 15659551 10.1073/pnas.0408709102 PMC547855

[19] Ehrenreich IM, Torabi N, Jia Y, Kent J, Martis S, et al (2010) Dissection of genetically complex traits with extremely large pools of yeast segregants. *Nature* 464:1039 10.1038/nature08923 20393561 PMC2862354

[20] Burch CL, Chao L (2004) Epistasis and its relationship to canalization in the RNA virus phi 6. *Genetics* 167:559 15238511 10.1534/genetics.103.021196 PMC1470902

[21] Weinreich DM, Watson RA, Chao L (2005) Perspective: Sign epistasis and genetic constraint on evolutionary trajectories. *Evolution* 59:1165 16050094

[22] Poelwijk FJ, Kiviet DJ, Weinreich DM, Tans SJ (2007) Empirical fitness landscapes reveal accessible evolutionary paths. *Nature* 445:383 17251971 10.1038/nature05451

[23] Poelwijk FJ, Tănase-Nicola S, Kiviet DJ, Tans SJ (2011) Reciprocal sign epistasis is a necessary condition for multi-peaked fitness landscapes. *J Theor Biol* 272:141 10.1016/j.jtbi.2010.12.015 21167837

[24] Lozovsky ER, Chookajorn T, Brown KM, Imwong M, Shaw PJ, et al (2009) Stepwise acquisition of pyrimethamine resistance in the malaria parasite. *Proc Natl Acad Sci USA* 106:12025 10.1073/pnas.0905922106 19587242 PMC2715478

[25] Maharjan RP, Ferenci T (2013) Epistatic interactions determine the mutational pathways and coexistence of lineages in clonal Escherichia coli populations. *Evolution* 67:2762 10.1111/evo.12137 24033182

[26] Draghi JA, Plotkin JB (2013) Selection biases the prevalence and type of epistasis along adaptive trajectories. *Evolution* 67:3120 10.1111/evo.12192 24151997

[27] VanderSluis B, Bellay J, Musso G, Costanzo M, Papp B, et al (2010) Genetic interactions reveal the evolutionary trajectories of duplicate genes. *Mol Syst Biol* 6:429 10.1038/msb.2010.82 21081923 PMC3010121

[28] Natarajan C, Inoguchi N, Weber RE, Fago A, Moriyama H, et al (2013) Epistasis among adaptive mutations in deer mouse hemoglobin. *Science* 340:1324 10.1126/science.1236862 23766324 PMC4409680

[29] Gong LI, Suchard MA, Bloom JD (2013) Stability-mediated epistasis constrains the evolution of an influenza protein. *eLife* 2:e00631 10.7554/eLife.00631 23682315 PMC3654441

[30] Ashworth A, Lord C, Reis-Filho J (2011) Genetic interactions in cancer progression and treatment. *Cell* 145:30 10.1016/j.cell.2011.03.020 21458666

[31] Chakravarti A, Clark AG, Mootha VK (2013) Distilling pathophysiology from complex disease genetics. *Cell* 155:21 10.1016/j.cell.2013.09.001 24074858 PMC4244836

[32] Leiserson MDM, Eldridge JV, Ramachandran S, Raphael BJ (2013) Network analysis of GWAS data. *Curr Opin Genet Dev* 23:602 10.1016/j.gde.2013.09.003 24287332 PMC3867794

[33] Hinkley T, Martins J, Chappey C, Haddad M, Stawiski E, et al (2011) A systems analysis of mutational effects in HIV-1 protease and reverse transcriptase. *Nat Genet* 43:487 10.1038/ng.795 21441930

[34] Combarros O, Cortina-Borja M, Smith AD, Lehmann DJ (2009) Epistasis in sporadic Alzheimer’s disease. *Neurobiol Aging* 30:1333 10.1016/j.neurobiolaging.2007.11.027 18206267

[35] Fitzgerald JB, Schoeberl B, Nielsen UB, Sorger PK (2006) Systems biology and combination therapy in the quest for clinical efficacy. *Nat Chem Biol* 2:458 16921358 10.1038/nchembio817

[36] Fu W, O’Connor TD, Akey JM (2013) Genetic architecture of quantitative traits and complex diseases. *Curr Opin Genet Dev* 23:678 10.1016/j.gde.2013.10.008 24287334 PMC6764439

[37] Wang X, Fu AQ, McNerney ME, White KP (2014) Widespread genetic epistasis among cancer genes. *Nature Comm* 5:4828 10.1038/ncomms5828 25407795

[38] Chen J, Stites WE (2001) Higher-order packing interactions in triple and quadruple mutants of staphylococcal nuclease. *Biochemistry* 40:14012 11705393 10.1021/bi011269d

[39] Frisch C, Schreiber G, Johnson CM, Fersht AR (1997) Thermodynamics of the interaction of barnase and barstar: changes in free energy versus changes in enthalpy on mutation. *J Mol Biol* 267:696 9126847 10.1006/jmbi.1997.0892

[40] Jiang C, Hwang YT, Wang G, Randell JCW, Coen DM, et al (2007) Herpes simplex virus mutants with multiple substitutions affecting DNA binding of UL42 are impaired for viral replication and DNA synthesis. *J Virol* 81:12077 17715219 10.1128/JVI.01133-07 PMC2168780

[41] Natarajan M, Lin KM, Hsueh RC, Sternweis PC, Ranganathan R (2006) A global analysis of cross-talk in a mammalian cellular signalling network. *Nat Cell Biol* 8:571 16699502 10.1038/ncb1418

[42] Weinreich DM, Delaney NF, Depristo MA, Hartl DL (2006) Darwinian evolution can follow only very few mutational paths to fitter proteins. *Science* 312:111 16601193 10.1126/science.1123539

[43] Aita T, Iwakura M, Husimi Y (2001) A cross-section of the fitness landscape of dihydrofolate reductase. *Protein Eng* 14:633 11707608 10.1093/protein/14.9.633

[44] Kinney JB, Murugan A, Callan CG, Cox EC (2010) Using deep sequencing to characterize the biophysical mechanism of a transcriptional regulatory sequence. *Proc Natl Acad Sci USA* 107:9158 10.1073/pnas.1004290107 20439748 PMC2889059

[45] Weinreich DM, Lan Y, Wylie CS, Heckendorn RB (2013) Should evolutionary geneticists worry about higher-order epistasis? *Curr Opin Genet Dev* 23:700 10.1016/j.gde.2013.10.007 24290990 PMC4313208

[46] Szendro IG, Schenk MF, Franke J, Krug J, de Visser JAGM (2013) Quantitative analyses of empirical fitness landscapes. *J Stat Mech* 2013:P01005.

[47] Weinberger DE (1991) Fourier and Taylor series on fitness landscapes. *Biol Cybern* 65:321

[48] Stadler PF (2002) Spectral landscape theory In: *Evolutionary Dynamics—Exploring the Interplay of Selection*, *Neutrality*, *Accident*, *and Function*, pages 231–272. Oxford University Press.

[49] Stadler PF (2002) Fitness landscapes In: *Biological Evolution and Statistical Physics*, pages 187–207, Springer-Verlag, Berlin

[50] Neidhart J, Szendro IG, Krug J (2013) Exact Results for Amplitude Spectra of Fitness Landscapes *Journal of Theoretical Biology* 332:218 10.1016/j.jtbi.2013.05.002 23685065

[51] Horovitz A, Fersht AR (1990) Strategy for analysing the co-operativity of intramolecular interactions in peptides and proteins. *J Mol Biol* 214:613 2388258 10.1016/0022-2836(90)90275-Q

[52] Horovitz A (1996) Double-mutant cycles: a powerful tool for analyzing protein structure and function. *Fold Des* 1:R121 9080186 10.1016/S1359-0278(96)00056-9

[53] Horovitz A, Fersht AR (1992) Co-operative interactions during protein folding. *J Mol Biol* 224:733 1569552 10.1016/0022-2836(92)90557-z

[54] Goldberg D (1989) Genetic Algorithms and Walsh Functions: Part I, A Gentle Introduction. *Complex Systems* 3:129.

[55] Beer T (1981) Walsh transforms. *American Journal of Physics* 49:466.

[56] Stoffer DS (1991) Walsh-Fourier analysis and its statistical applications. *Journal of the American Statistical Association* 86:461.

[57] McLaughlin RN, Poelwijk FJ, Raman A, Gosal WS, Ranganathan R (2012) The spatial architecture of protein function and adaptation. *Nature* 491:138 10.1038/nature11500 23041932 PMC3991786

[58] Niethammer M, Valtschanoff JG, Kapoor TM, Allison DW, Weinberg RJ, Craig AM, et al (1998) CRIPT, a novel postsynaptic protein that binds to the third PDZ domain of PSD-95/SAP90. *Neuron* 20:693 9581762 10.1016/s0896-6273(00)81009-0

[59] Stiffler MA, Chen JR, Grantcharova VP, Lei Y, Fuchs D, Allen JE, et al (2007) PDZ domain binding selectivity is optimized across the mouse proteome. *Science* 317:364 17641200 10.1126/science.1144592 PMC2674608

[60] Sadovsky Y, Yifrach O (2007) Principles underlying energetic coupling along an allosteric communication trajectory of a voltage-activated K+ channel. *Proc Natl Acad Sci USA* 104:19813 18077413 10.1073/pnas.0708120104 PMC2148381

[61] Yifrach O, MacKinnon R (2002) Energetics of pore opening in a voltage-gated K+ channel. *Cell* 111:231 12408867 10.1016/s0092-8674(02)01013-9

[62] Hartl DL (2014) What can we learn from fitness landscapes? *Curr Opin Microbiol* 21:51 10.1016/j.mib.2014.08.001 25444121 PMC4254422

[63] Poelwijk FJ, de Vos MGJ, Tans SJ (2011) Tradeoffs and optimality in the evolution of gene regulation. *Cell* 146:462 10.1016/j.cell.2011.06.035 21802129

[64] Sierpinski W (1915) Sur une courbe dont tout point est un point de ramification. *CR hebd Acad Science Paris* 160:302.

[65] Hastie T, Tibshirani R, Friendman J (2009) The Elements of Statistical Learning, 2nd ed New York: Springer Publishing. Springer Series in Statistics.

[66] Tibshirani R (1996) Regression shrinkage and selection via the Lasso. *J Roy Stat Soc*: *Ser B* 58:267.

[67] Otwinowski J, Plotkin JB (2014) Inferring fitness landscapes by regression produces biased estimates of epistasis. *Proc Natl Acad Sci USA* 111:E2301 10.1073/pnas.1400849111 24843135 PMC4050575

[68] Shi L, Kay LE (2014) Tracing an allosteric pathway regulating the activity of the HslV protease. *Proc Natl Acad Sci USA* 111:2140 10.1073/pnas.1318476111 24469799 PMC3926032

[69] Ruschak AM, Kay LE (2012) Proteasome allostery as a population shift between interchanging conformers. *Proc Natl Acad Sci USA* 109:E3454 10.1073/pnas.1213640109 23150576 PMC3528551

[70] Luque I, Leavitt SA, Freire E (2002) The linkage between protein folding and functional cooperativity: two sides of the same coin? *Ann Rev Biophys Biomol Struct* 31:235. 11988469 10.1146/annurev.biophys.31.082901.134215

[71] Halabi N, Rivoire O, Leibler S, Ranganathan R (2009) Protein sectors: evolutionary units of three-dimensional structure. *Cell* 138: 774 10.1016/j.cell.2009.07.038 19703402 PMC3210731

[72] Socolich M, Lockless SW, Russ WP, Lee HL, Gardner K, Ranganathan R (2005) Evolutionary information for specifying a protein fold. *Nature* 437:512 16177782 10.1038/nature03991

[73] Russ WP, Lowery DM, Mishra P, Yaffe MB, Ranganathan R (2005) Natural-like function in artificial WW domains. *Nature* 437:579 16177795 10.1038/nature03990

[74] Kirschner M, Gerhart J (1998) Evolvability. *Proc Natl Acad Sci USA* 95:8420 9671692 10.1073/pnas.95.15.8420 PMC33871

[75] Candès EJ, Wakin MB (2008) An introduction to compressive sampling. *IEEE Signal Proc Mag* 25:21.

[76] Candès EJ, Wakin MB, Boyd SP (2008) Enhancing sparsity by reweighted *l1* minimization. *J Fourier Anal Appl* 14:877.

[77] Casari G, Sander C, Valencia A (1995) A method to predict functional residues in proteins. *Nature Structural Biology* 2:171 7749921 10.1038/nsb0295-171

[78] Lapedes AS, Giraud BG, Jarzynski C (2002) Using sequence alignments to predict protein structure and stability with high accuracy. LANL preprint: http://library.lanl.gov/cgi-bin/getfile?01038177.pdf

[79] Burger L, van Nimwegen E (2008) Accurate prediction of protein-protein interactions from sequence alignments using a Bayesian method. *Mol Syst Biol* 4:165 10.1038/msb4100203 18277381 PMC2267735

[80] Weigt M, White RA, Szurmant H, Hoch JA, Hwa T (2009) Identification of direct residue contacts in protein–protein interaction by message passing. *Proc Natl Acad Sci* 106:67 10.1073/pnas.0805923106 19116270 PMC2629192

[81] Morcos F, Pagnani A, Lunt B, Bertolino A, Marks DS, Sander C, Zecchina R, Onuchic JN, Hwa T, Weigt M (2011) Direct-coupling analysis of residue coevolution captures native contacts across many protein families. *Proc Natl Acad Sci* 108:E1293 10.1073/pnas.1111471108 22106262 PMC3241805

[82] Marks DS, Hopf T, Sander C (2012) Protein structure prediction from sequence variation. *Nat*. *Biotechnol* 30:1072 10.1038/nbt.2419 23138306 PMC4319528

[83] Skerker JM, Perchuk BS, Siryaporn A, Lubin EA, Ashenberg O, Goulian M, Laub MT (2008) Rewiring the specificity of two-component signal transduction systems. *Cell* 133:1043 10.1016/j.cell.2008.04.040 18555780 PMC2453690

[84] Lockless SW, Ranganathan R (1999) Evolutionarily conserved pathways of energetic connectivity in protein families. *Science* 286:295 10514373 10.1126/science.286.5438.295

[85] Süel G, Lockless SW, Wall MA, Ranganathan R (2003) Evolutionarily conserved networks of residues mediate allosteric communication in proteins. *Nat Struct Biol* 10:59 12483203 10.1038/nsb881

[86] Zaremba SM, Gregoret LM (1999) Context-dependence of amino acid residue pairing in antiparallel β-sheets. *J Mol Biol* 291:463 10438632 10.1006/jmbi.1999.2961

[87] Shepherd TR, Hard RL, Murray AM, Pei D, Fuentes EJ (2011) Distinct ligand specificity of the Tiam1 and Tiam2 PDZ domains. *Biochemistry* 50:1296 10.1021/bi1013613 21192692 PMC3059893

[88] Hidalgo P, MacKinnon R (1995) Revealing the architecture of a K + channel pore through mutant cycles with a peptide inhibitor. *Science* 268:307 7716527 10.1126/science.7716527

[89] Carter PJ, Winter G, Wilkinson AJ, Fersht AR (1984) The use of double mutants to detect structural changes in the active site of the tyrosyl-tRNA synthetase (*Bacillus stearothermophilus*). *Cell* 38:835 6488318 10.1016/0092-8674(84)90278-2

[90] Ranganathan R, Lewis JH, MacKinnon R (1996) Spatial localization of the K+ channel selectivity filter by mutant cycle-based structure analysis. *Neuron* 16:131 8562077 10.1016/s0896-6273(00)80030-6

[91] Otwinowski J, Nemenman I (2013) Genotype to phenotype mapping and the fitness landscape of the E. coli lac promoter. *PLoS ONE* 8:e61570 10.1371/journal.pone.0061570 23650500 PMC3641078

