# Supplementary material for: The Context-Dependence of Mutations: A Linkage of Formalisms
Source: PLoS Comput Biol. 2016 Jun 23;12(6):e1004771. doi: 10.1371/journal.pcbi.1004771 (PMC4919011; doi:10.1371/journal.pcbi.1004771)
Supplement: S1 Text — (PDF) [file pcbi.1004771.s001.pdf]

S1 Text. Additional proofs; expressing epistasis operators as Hadamard transforms.

A. Expressing the biochemical epistasis operator $\boldsymbol{G}$ as a Hadamard transform

$\boldsymbol{G} = \boldsymbol{X}^{-1} = \boldsymbol{V}\boldsymbol{X}^T\boldsymbol{H}$ (Eq. 17 in the main text)

First we write the different matrix operators in their recursive form, and then proceed by induction. We have for the recursive form of $\boldsymbol{X}$:

$$\boldsymbol{X}_{n+1} = \begin{pmatrix} \boldsymbol{X}_n & 0 \\ \boldsymbol{X}_n & \boldsymbol{X}_n \end{pmatrix} \quad \text{with} \quad \boldsymbol{X}_0 = 1$$

In order to find the generative function for the inverse $\boldsymbol{X}^{-1}$ we can write $\boldsymbol{X}_{n+1}\boldsymbol{X}^{-1}_{n+1} = \mathbb{I}$:

$$\begin{pmatrix} \boldsymbol{X}_n & 0 \\ \boldsymbol{X}_n & \boldsymbol{X}_n \end{pmatrix} \boldsymbol{X}^{-1}_{n+1} = \begin{pmatrix} \mathbb{I} & 0 \\ 0 & \mathbb{I} \end{pmatrix},$$

which we can solve by Gauss-Jordan elimination:

$$\left(\begin{array}{cc|cc} \boldsymbol{X}_n & 0 & \mathbb{I} & 0 \\ \boldsymbol{X}_n & \boldsymbol{X}_n & 0 & \mathbb{I} \end{array}\right) \Rightarrow$$

$$\left(\begin{array}{cc|cc} \mathbb{I} & 0 & \boldsymbol{X}^{-1}_n & 0 \\ \mathbb{I} & \mathbb{I} & 0 & \boldsymbol{X}^{-1}_n \end{array}\right) \Rightarrow \left(\begin{array}{cc|cc} \mathbb{I} & 0 & \boldsymbol{X}^{-1}_n & 0 \\ 0 & \mathbb{I} & -\boldsymbol{X}^{-1}_n & \boldsymbol{X}^{-1}_n \end{array}\right)$$

hence we have for the inverse of $\boldsymbol{X}$:

$$\boldsymbol{X}^{-1}_{n+1} = \begin{pmatrix} \boldsymbol{X}^{-1}_n & 0 \\ -\boldsymbol{X}^{-1}_n & \boldsymbol{X}^{-1}_n \end{pmatrix} \quad \text{with} \quad \boldsymbol{X}^{-1}_0 = 1$$

Which is identical to the recursive form for $\boldsymbol{G}$:

$$\boldsymbol{G}_{n+1} = \begin{pmatrix} \boldsymbol{G}_n & 0 \\ -\boldsymbol{G}_n & \boldsymbol{G}_n \end{pmatrix}$$

We further have:

$$\boldsymbol{H}_{n+1} = \begin{pmatrix} \boldsymbol{H}_n & \boldsymbol{H}_n \\ \boldsymbol{H}_n & -\boldsymbol{H}_n \end{pmatrix} \quad \text{with} \quad \boldsymbol{H}_0 = 1$$

and $\boldsymbol{V}_{n+1} = \begin{pmatrix} \frac{1}{2}\boldsymbol{V}_n & 0 \\ 0 & -\boldsymbol{V}_n \end{pmatrix}$ with $\boldsymbol{V}_0 = 1$

With the above relations we can derive the equality in the main text expressing $\boldsymbol{G}$ as a Hadamard transform:

$$\boldsymbol{G}_{n} = \boldsymbol{X}_{n}^{-1} = \boldsymbol{V}_{n}\boldsymbol{X}_{n}^{T}\boldsymbol{H}_{n}$$

For $n = 0$ the statement is trivial. We now show by induction that this relation holds for all n.

$$\boldsymbol{G}_{n+1} = \begin{pmatrix} \boldsymbol{G}_n & 0 \\ -\boldsymbol{G}_n & \boldsymbol{G}_n \end{pmatrix} = \begin{pmatrix} \boldsymbol{V}_{n}\boldsymbol{X}_{n}^{T}\boldsymbol{H}_n & 0 \\ -\boldsymbol{V}_{n}\boldsymbol{X}_{n}^{T}\boldsymbol{H}_n & \boldsymbol{V}_{n}\boldsymbol{X}_{n}^{T}\boldsymbol{H}_n \end{pmatrix}$$

$$= \begin{pmatrix} \frac{1}{2}\boldsymbol{V}_n & 0 \\ 0 & -\boldsymbol{V}_n \end{pmatrix} \begin{pmatrix} 2\boldsymbol{X}_{n}^{T}\boldsymbol{H}_n & 0 \\ \boldsymbol{X}_{n}^{T}\boldsymbol{H}_n & -\boldsymbol{X}_{n}^{T}\boldsymbol{H}_n \end{pmatrix}$$

$$= \begin{pmatrix} \frac{1}{2}\boldsymbol{V}_n & 0 \\ 0 & -\boldsymbol{V}_n \end{pmatrix} \begin{pmatrix} \boldsymbol{X}_{n}^{T} & \boldsymbol{X}_{n}^{T} \\ 0 & \boldsymbol{X}_{n}^{T} \end{pmatrix} \begin{pmatrix} \boldsymbol{H}_n & \boldsymbol{H}_n \\ \boldsymbol{H}_n & -\boldsymbol{H}_n \end{pmatrix}$$

$$= \boldsymbol{V}_{n+1}\boldsymbol{X}_{n+1}^{T}\boldsymbol{H}_{n+1} \qquad \text{QED}$$

B. Expressing the regression operator as a Hadamard transform

$$\boldsymbol{Q}\left(\hat{\boldsymbol{X}}^{T}\hat{\boldsymbol{X}}\right)^{-1}\hat{\boldsymbol{X}}^{T} = \boldsymbol{V}\boldsymbol{X}^{T}\boldsymbol{S}\boldsymbol{H} \qquad \text{(Eq. 18 in the main text)}$$

We will use $\hat{\boldsymbol{X}} = \boldsymbol{X}\boldsymbol{Q}$ and $\boldsymbol{S} = \boldsymbol{Q}\boldsymbol{Q}^{T}$ as defined in the main text.

For the right-hand side we can write

$$\boldsymbol{V}\boldsymbol{X}^{T}\boldsymbol{S}\boldsymbol{H} = \frac{1}{2^n}\boldsymbol{V}\boldsymbol{X}^{T}\left(\boldsymbol{H}\boldsymbol{H}\right)\boldsymbol{S}\boldsymbol{H}$$

where we used $\boldsymbol{H}_{n}^{2} = 2^{n}\mathbb{I}_{n}$, which can be proven straightforwardly by induction using the generative function for $\boldsymbol{H}$.
Rearranging and using $\boldsymbol{X}^{-1} = \boldsymbol{V}\boldsymbol{X}^{T}\boldsymbol{H}$, we obtain

$$\boldsymbol{V}\boldsymbol{X}^{T}\boldsymbol{S}\boldsymbol{H} = \frac{1}{2^n}\boldsymbol{X}^{-1}\left(\boldsymbol{H}\boldsymbol{S}\boldsymbol{H}\right)$$

We thus have to prove

$$\boldsymbol{Q}\left(\hat{\boldsymbol{X}}^{T}\hat{\boldsymbol{X}}\right)^{-1}\hat{\boldsymbol{X}}^{T} = \frac{1}{2^n}\boldsymbol{X}^{-1}\left(\boldsymbol{H}\boldsymbol{S}\boldsymbol{H}\right)$$

Left-multiplying both sides by $\hat{\boldsymbol{X}}^{T}\boldsymbol{X}$ (mind the hat is only on the first operator) and right-multiplying by $\boldsymbol{H}$ we are left to prove

$$\hat{\boldsymbol{X}}^{T}\boldsymbol{H} = \hat{\boldsymbol{X}}^{T}\boldsymbol{H}\boldsymbol{S}$$

Left-multiplication by $\boldsymbol{Q}$ yields

$$\boldsymbol{S}\boldsymbol{X}^{T}\boldsymbol{H} = \boldsymbol{S}\boldsymbol{X}^{T}\boldsymbol{H}\boldsymbol{S}$$

which, again using the relation we proved in section A above, can be rewritten as

$$\boldsymbol{S}\boldsymbol{V}^{-1}\boldsymbol{X}^{-1} = \boldsymbol{S}\boldsymbol{V}^{-1}\boldsymbol{X}^{-1}\boldsymbol{S}$$

or

$$\boldsymbol{S}\boldsymbol{X}^{-1} = \boldsymbol{S}\boldsymbol{X}^{-1}\boldsymbol{S}$$

given the commutative properties of diagonal matrices $\boldsymbol{S}$ and $\boldsymbol{V}^{-1}$.

This equality indicates that setting certain rows of $\boldsymbol{X}^{-1}$ to zero (left-hand side) is the same as setting both those rows and corresponding columns of $\boldsymbol{X}^{-1}$ to zero (right-hand side). This is obviously not true for every set of rows and columns, and needs more discussion.

We can prove this iteratively starting at regression to order $n-1$ and going down to lower order. If regression is done to order $n-1$, this means that only the last row of $\boldsymbol{X}^{-1}$ is set to zero, and by construction of $\boldsymbol{X}^{-1}$ (see above) the last column only has a non-zero element in this row. This means that in this case the equality is correct. Another way to see this is looking at matrix $\boldsymbol{G}$ for $n = 3$ in its explicit representation in the main text (here $\boldsymbol{G}$ being identified with $\boldsymbol{X}^{-1}$) and noting that the highest order epistatic term λ_{111} is the only one that receives a contribution from the highest order (n) mutant term y_{111}.

Next, if regression is performed instead to order $n-2$, not only the last row of $\boldsymbol{X}^{-1}$ is set to zero, but also the rows corresponding to $n-1$ order mutants. Analogously to above, the only terms in the vector $\bar{\boldsymbol{\lambda}}$ that receive contributions from the $n-1$ order mutants are the ones in the rows corresponding to $n-1$ order of epistasis (since the row corresponding to n^{th} order is already set to zero), meaning that their corresponding column again has only one non-zero element. Hence setting these rows to zero will directly set their corresponding column to zero, and the equality holds.

And so forth for regression to order $n-3$, etc., etc.

QED
