# Supplementary material for: The Context-Dependence of Mutations: A Linkage of Formalisms
Source: PLoS Comput Biol. 2016 Jun 23;12(6):e1004771. doi: 10.1371/journal.pcbi.1004771 (PMC4919011; doi:10.1371/journal.pcbi.1004771)
Supplement: S2 Text — (PDF) [file pcbi.1004771.s002.pdf]

S2 Text. Error propagation in biochemical and background-averaged epistasis

Here we will discuss the difference in error propagation between background-averaged and biochemical epistasis. In the definition of the biochemical epistasis the contribution of each order n is a difference of two terms of order $n-1$. If we assume errors on all measurements $\bar{\boldsymbol{y}}$ are uncorrelated and of equal size, we can expect this to translate into an increase of the uncertainty by a factor of $\sqrt{2}$ for each order. By the definition of the background-averaged epistasis the uncertainty grows faster, because not only is each higher order a sum of two lower orders, but it is also averaged over 2-fold fewer genetic backgrounds. This yields an increase of a factor $\sqrt{2} * \sqrt{2} = 2$ for every order, which is corroborated by Figure S1, where we plotted the widths of the histograms of the values of the epistatic contributions for a computer simulated dataset with $N = 14$ where $\bar{\boldsymbol{y}}$ is a constant with Gaussian noise of width $\sigma = 1$.

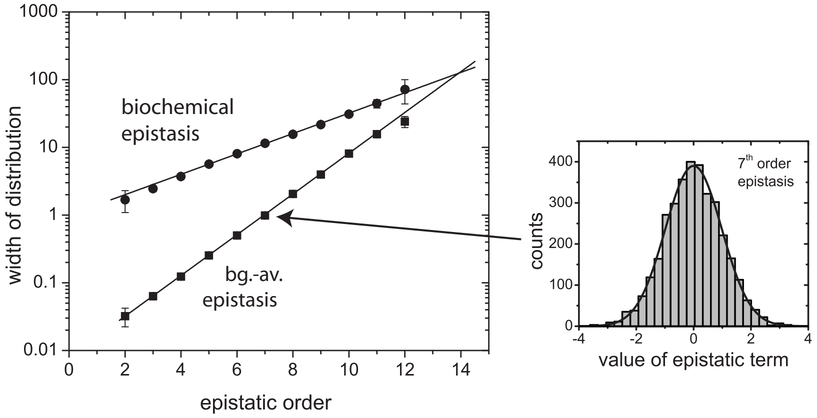

Figure S1. Propagation of errors in epistatic terms due to noise in the measured data. Plotted in the main graph are the widths (SD) of the histograms of epistatic terms of each order for a simulated flat dataset with $N = 14$ and a fixed Gaussian noise with $\sigma = 1$, for both biochemical and background-averaged epistasis. The inset on the right is an example of the histogram for the calculated 7th order background-averaged contributions. Straight lines in the main graph have the appropriate slopes to indicate an increase in uncertainty by a factor 2 (lower line) or a factor $\sqrt{2}$ (upper line) per order, respectively, and intersect at $N = 14$, in accordance with the expectations of propagation of errors. The intercept of the fit of the biochemical epistasis with the y-axis corresponds to the standard deviation of the noise of the dataset $\sigma = 1$.

More formally, we can write the vector of uncertainties in epistatic quantity $\bar{\boldsymbol{\omega}}$ as the square root of the sum of squares of the uncertainties in the measured quantities y_i. This can be written in matrix notation as:

$$\overline{\boldsymbol{\delta\omega}} = (\boldsymbol{\Omega}_{\text{epi}} \circ \boldsymbol{\Omega}_{\text{epi}} \ \overline{\boldsymbol{\delta y}} \circ \overline{\boldsymbol{\delta y}})^{1/2} \tag{s1}$$

where $\overline{\boldsymbol{\delta y}}$ is the vector of uncertainties in $\bar{\boldsymbol{y}}$ and $\circ$ is the element-wise product. If the uncertainties in y_i are all of equal size σ, we can replace $\overline{\boldsymbol{\delta y}} \circ \overline{\boldsymbol{\delta y}}$ by $\sigma^2 \bar{\mathbf{1}}$, where $\bar{\mathbf{1}}$ is a column vector of length 2^n with all entries being 1's.

For the biochemical view of epistasis Eq. s1 then reduces to

$$\overline{\boldsymbol{\delta\lambda}} = \sigma \left((\boldsymbol{G} \circ \boldsymbol{G}) \ \bar{\mathbf{1}} \right)^{1/2} = \sigma \left(\sum_j (\boldsymbol{G} \circ \boldsymbol{G})_{ij} \right)^{1/2} = \sigma \left[(\sqrt{2})^{q_i} \right] \tag{s2}$$

where [.] represents a column vector and q_i is the order of the epistatic contribution in row i. Uncertainties thus grow with a factor $\sqrt{2}$ per order.

For the background-averaged epistasis assuming equal uncertainties leads to

$$\overline{\boldsymbol{\delta\varepsilon}} = \sigma \left((\boldsymbol{VH}) \circ (\boldsymbol{VH}) \ \bar{\mathbf{1}} \right)^{1/2} = \sigma \left(2^n \sum_j (\boldsymbol{V} \circ \boldsymbol{V})_{ij} \right)^{1/2} = \frac{\sigma}{2^{n/2}} \left[2^{q_i} \right] \tag{s3}$$

In this case, the uncertainty can be seen to grow with a factor 2 per order. At the highest order $q_i = n$, where biochemical and background-averaged epistasis are identical, the uncertainties are obviously identical too.
