# Supplementary material for: The Context-Dependence of Mutations: A Linkage of Formalisms
Source: PLoS Comput Biol. 2016 Jun 23;12(6):e1004771. doi: 10.1371/journal.pcbi.1004771 (PMC4919011; doi:10.1371/journal.pcbi.1004771)
Supplement: S3 Text — (PDF) [file pcbi.1004771.s003.pdf]

S3 Text. Analysis of epistasis in a K^+ channel

In Table 2 in the main text we compare biochemical epistasis and background-averaged epistasis for a complete 4th-order combinatorial landscape of mutations in the pore of the voltage-activated *Shaker* K^+ channel. Data is obtained from [1] and [2]. Mutant potassium channels were expressed in *Xenopus* oocytes and the potassium conductance was measured as a function of cell membrane depolarization. The S-shaped activation curve was parametrized by two parameters: the voltage at half-activation and a parameter that is proportional to the slope at half-activation. These parameters were correlated and in a two-state model for pore opening the product of these parameters is directly proportional to the free energy difference of pore opening. Hence, this parameter, designated here as ΔG_{open}, is directly mappable on a thermodynamic state variable and the null hypothesis for epistasis is the *additivity* in this parameter.

Table 2 shows the calculated ΔG_{open} for a set of four positions (A391, E395, A465, and T469) that were deemed to be part of the allosteric trajectory of channel opening. Along this trajectory inter-residue interactions are necessary for a concerted conformational change in the channel, and therefore a high cooperativity between these residues was expected and demonstrated [2]. Here we calculated the biochemical epistasis (as in ref. [2]) alongside the background-averaged epistasis (Table 2) using the formalisms described in the main text, with $n = 4$. Sadovsky and Yifrach's [2] argument for the high-cooperativity in the selected residues is based on comparison of three-way epistatic terms of on-trajectory mutations to three-way terms for triplets containing an off-trajectory mutation. Here, based on the values for the background-averaged epistatic terms we can give an additional argument: all terms (except for a trivial zeroth order term) are small compared to the maximum (fourth) order term. This becomes even clearer when we take the mean of the absolute values of the terms within each order (data in Table 3). This signature strongly suggests that all four positions act as one cooperative unit in this protein. Interestingly, as one of the objectives of ref. [2] was to delineate the functionally conserved boundaries of the allosteric trajectories, we propose here that background-averaged epistasis is a natural way to find conserved cooperative units in proteins.

References

1. Yifrach O, MacKinnon R (2002) Energetics of pore opening in a voltage-gated K+ channel. *Cell* **111**:231.

2. Sadovsky Y, Yifrach O (2007) Principles underlying energetic coupling along an allosteric communication trajectory of a voltage-activated K+ channel. *Proc Natl Acad Sci USA* **104**:19813.
