# Supplementary material for: The Context-Dependence of Mutations: A Linkage of Formalisms
Source: PLoS Comput Biol. 2016 Jun 23;12(6):e1004771. doi: 10.1371/journal.pcbi.1004771 (PMC4919011; doi:10.1371/journal.pcbi.1004771)
Supplement: S4 Text — (PDF) [file pcbi.1004771.s004.pdf]

S4 Text. Fourier and Taylor decompositions as a result of genotype definition

Here we explicitly discuss the relation between a Taylor and Fourier expansion of the fitness/phenotypic landscape $\bar{\boldsymbol{y}}$ and the definition of biochemical and background-averaged epistasis. The Taylor expansion of a fitness landscape is given (e.g. [1]) as

$$y_g = b_0 + \sum_{i=1}^{n} b_i g_i + \sum_{i<j}^{n} b_{ij} g_i g_j + \sum_{i<j<k}^{n} b_{ijk} g_i g_j g_k + ... \tag{s4}$$

where the genotype is specified as a string of g_i being 0's and 1's, for either the wild-type amino acid or the mutant amino acid. The Fourier expansion can be found rather simply by reparametrizing the genotype as -1/+1, hence by substitution of

$$g_i = \frac{\sigma_i + 1}{2}. \tag{s5}$$

Working this out explicitly for $N = 3$ yields:

$$y_\sigma = b_0 + \sum_{i=1}^{3} b_i \Big(\frac{\sigma_i+1}{2}\Big) + \sum_{i<j}^{3} b_{ij} \Big(\frac{\sigma_i+1}{2}\Big)\Big(\frac{\sigma_j+1}{2}\Big) + \sum_{i<j<k}^{3} b_{ijk} \Big(\frac{\sigma_i+1}{2}\Big)\Big(\frac{\sigma_j+1}{2}\Big)\Big(\frac{\sigma_k+1}{2}\Big) \tag{s6}$$

Rearranging terms and gathering the zeroth-, first-, second-, and third-order coefficients for σ_i, and renaming those coefficients as e's, we obtain:

$$\begin{pmatrix} e_{000} \\ e_{001} \\ e_{010} \\ e_{011} \\ e_{100} \\ e_{101} \\ e_{110} \\ e_{111} \end{pmatrix} = \frac{1}{8} * \begin{pmatrix} 8 & 4 & 4 & 2 & 4 & 2 & 2 & 1 \\ 0 & 4 & 0 & 2 & 0 & 2 & 0 & 1 \\ 0 & 0 & 4 & 2 & 0 & 0 & 0 & 1 \\ 0 & 0 & 0 & 2 & 0 & 0 & 0 & 1 \\ 0 & 0 & 0 & 0 & 4 & 2 & 2 & 1 \\ 0 & 0 & 0 & 0 & 0 & 2 & 0 & 1 \\ 0 & 0 & 0 & 0 & 0 & 0 & 2 & 1 \\ 0 & 0 & 0 & 0 & 0 & 0 & 0 & 1 \end{pmatrix} * \begin{pmatrix} b_{000} \\ b_{001} \\ b_{010} \\ b_{011} \\ b_{100} \\ b_{101} \\ b_{110} \\ b_{111} \end{pmatrix} \tag{s7}$$

In our manuscript we define the background-averaged epistasis

$$\bar{\boldsymbol{\varepsilon}} = \boldsymbol{V}\boldsymbol{H}\bar{\boldsymbol{y}} \tag{s8}$$

and the biochemical epistasis

$$\bar{\boldsymbol{\lambda}} = \boldsymbol{V}\boldsymbol{X}^T\boldsymbol{H}\bar{\boldsymbol{y}} \tag{s9}$$

which yields for the relation between the epistatic terms

$$\bar{\boldsymbol{\varepsilon}} = \boldsymbol{V}(\boldsymbol{X}^T)^{-1}\ \boldsymbol{V}^{-1}\bar{\boldsymbol{\lambda}} \tag{s10}$$

This is, apart from an order-dependent weighting factor Z, identical to Eq. s7, which can be rewritten as

$$\bar{\boldsymbol{e}} = Z\ \boldsymbol{V}(\boldsymbol{X}^T)^{-1}\ \boldsymbol{V}^{-1}\bar{\boldsymbol{b}} \tag{s11}$$

where Z is a diagonal matrix with elements $z_{ii} = 1/2^{q_i}$ and q_i is the order of the epistatic contribution in row i.

References

1. Weinberger DE (1991) Fourier and Taylor series on fitness landscapes. *Biol Cybern* **65**:321
