# Supplementary material for: The Context-Dependence of Mutations: A Linkage of Formalisms
Source: PLoS Comput Biol. 2016 Jun 23;12(6):e1004771. doi: 10.1371/journal.pcbi.1004771 (PMC4919011; doi:10.1371/journal.pcbi.1004771)
Supplement: S5 Text — (PDF) [file pcbi.1004771.s005.pdf]

S5 Text. Relation between Fourier decomposition and ANOVA

Here we discuss the connection between the Fourier decomposition of a fitness landscape and ANOVA (ANalysis Of VAriance), as noted by several authors (see e.g. [1]). ANOVA is a procedure commonly used in biology to asses the statistical significance of main effects and interactions by comparing variances between and among groups. This is done by decomposing the total variance of a variable into the sum of conditional variances due to the different effects and interactions that contribute to the variable. We will here discuss the mathematical similarities, but we do also note that the focus of ANOVA and the epistatic analysis presented here is usually slightly different: whereas ANOVA-based approaches are used to determine the significance of main effects and interactions, the objective of epistatic analysis is to infer the most likely value of epistasis at a given order.

To make the connection mathematically explicit, we start by formally writing the fitness landscape as a Fourier expansion over each of the genotype variables:

$$f(\bar{\boldsymbol{g}}) = \sum_{s_1, s_2, s_3, \ldots} \Gamma(\bar{\boldsymbol{s}}) \exp\left[\pi i \bar{\boldsymbol{s}} \cdot \bar{\boldsymbol{g}}\right] \tag{s12}$$

where $\bar{\boldsymbol{g}} \equiv \{g_1, g_2, ..., g_n\}$ represent the genotype and $f(\bar{\boldsymbol{g}})$ the fitness function. This can be written explicitly as:

$$f(\bar{\boldsymbol{g}}) = \Gamma(0,0,0,...) + \sum_{s_j} \Gamma(0,0,...,s_j,...)\, \mathrm{e}^{\pi i s_j g_j} + \sum_{s_j, s_k} \Gamma(0,0,...,s_j,...,s_k,...)\, \mathrm{e}^{\pi i (g_j s_j + g_k s_k)} + ... \tag{s13}$$

This expansion has the form:

$$f(\bar{\boldsymbol{g}}) = f_0 + \sum_j f_j (-1)^{s_j g_j} + \sum_{j<k} f_{jk} (-1)^{s_j g_j + s_k g_k} + ... \tag{s14}$$

with the terms being related to the Fourier coefficients as

$$f_0 = \Gamma(0,0,0,0,...);\;\; f_j = \Gamma(0,0,0,...,s_j,...); \mathrm{etc} \tag{s15}$$

The variables take on the values $s_j \in [-1, 1]$. For simplicity of notation, we scale the mean fitness $\langle f(\bar{\boldsymbol{g}}) \rangle = 0$. Then, the variance is proportional to the mean square of the fitness: $\mathrm{Var}(f) \equiv \mathcal{V} \propto \sum_{\bar{\boldsymbol{g}}} f^2(\bar{\boldsymbol{g}})$. Thus, we have

$$V \propto \sum_{\bar{\boldsymbol{g}}} \sum_{\bar{\boldsymbol{s}}, \bar{\boldsymbol{s}}'} \Gamma(\bar{\boldsymbol{s}}) \Gamma^*(\bar{\boldsymbol{s}}') e^{\pi i (\bar{\boldsymbol{s}} - \bar{\boldsymbol{s}}') \cdot \bar{\boldsymbol{g}}} \tag{s16}$$

Since the Fourier basis functions are orthonormal, the terms with $\bar{\boldsymbol{s}} \neq \bar{\boldsymbol{s}}'$ are zero, and the variance of the fitness decomposes similarly to the fitness:

$$\mathcal{V} \propto \sum_{\bar{\boldsymbol{g}}} f^2(\bar{\boldsymbol{g}}) = f_0^2 + \sum_j f_j^2 + \sum_{j<k} f_{jk}^2 + ... \tag{s17}$$

or

$$\mathcal{V} = \mathcal{V}_0 + \sum_j \mathcal{V}_j + \sum_{j<k} \mathcal{V}_{jk} + ... \tag{s18}$$

This expansion of the total variance is similar to that of an ANOVA model. To determine the significance of an effect or interaction, a statistic based on the ratios of variances between and within groups is calculated and compared to a standard distribution.

References

1. Szendro IG, Schenk MF, Franke J, Krug J, de Visser JAGM (2013) Quantitative analyses of empirical fitness landscapes. *J Stat Mech* **2013**:P01005.
