# Supplementary material for: The Context-Dependence of Mutations: A Linkage of Formalisms
Source: PLoS Comput Biol. 2016 Jun 23;12(6):e1004771. doi: 10.1371/journal.pcbi.1004771 (PMC4919011; doi:10.1371/journal.pcbi.1004771)
Supplement: S1 Fig — Plotted in the main graph are the widths (SD) of the histograms of epistatic terms of each order for a simulated flat dataset with N = 14 and a fixed Gaussian noise with σ = 1, for both biochemical and background-averaged epistasis. The inset on the right is an example of the histogram for the calculated 7th order background-averaged contributions. Straight lines in the main graph have the appropriate slopes to indicate an increase in uncertainty by a factor 2 (lower line) or a factor 2 (upper line) per order, respectively, and intersect at N = 14, in accordance with the expectations of propagation of errors. The intercept of the fit of the biochemical epistasis with the y-axis corresponds to the standard deviation of the noise of the dataset σ = 1. (PDF) [file pcbi.1004771.s006.pdf]

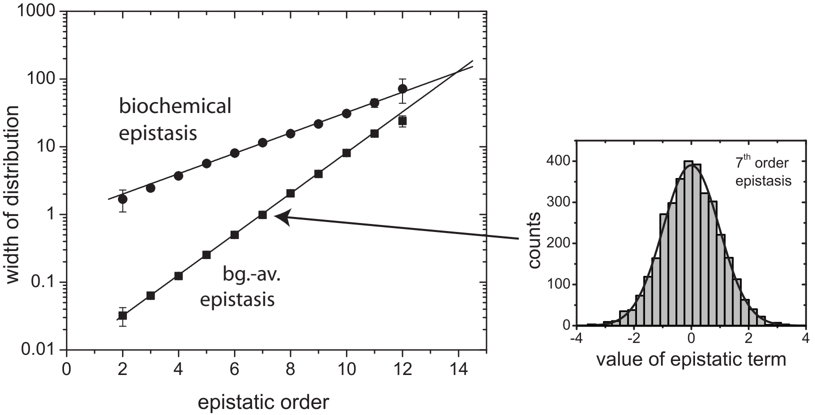

Figure S1. Propagation of errors in epistatic terms due to noise in the measured data. Plotted in the main graph are the widths (SD) of the histograms of epistatic terms of each order for a simulated flat dataset with $N = 14$ and a fixed Gaussian noise with $\sigma = 1$, for both biochemical and background-averaged epistasis. The inset on the right is an example of the histogram for the calculated 7th order background-averaged contributions. Straight lines in the main graph have the appropriate slopes to indicate an increase in uncertainty by a factor 2 (lower line) or a factor $\sqrt{2}$ (upper line) per order, respectively, and intersect at $N = 14$, in accordance with the expectations of propagation of errors. The intercept of the fit of the biochemical epistasis with the y-axis corresponds to the standard deviation of the noise of the dataset $\sigma = 1$.

identical, the uncertainties are obviously identical too.
